# Experience Does Not Equal Expertise in Recognizing Infrequent Incoming Gunfire: Neural Markers for Experience and Task Expertise at Peak Behavioral Performance

**DOI:** 10.1371/journal.pone.0115629

**Published:** 2015-02-06

**Authors:** Jason Samuel Sherwin, Jeremy Rodney Gaston

**Affiliations:** 1 Department of Ophthalmology, State University of New York, Downstate Medical Center, Brooklyn, NY United States of America; 2 Human Research and Engineering Directorate, U.S. Army Research Laboratory, Aberdeen Proving Ground, MD United States of America

## Abstract

For a soldier, decisions to use force can happen rapidly and sometimes lead to undesired consequences. In many of these situations, there is a rapid assessment by the shooter that recognizes a threat and responds to it with return fire. But the neural processes underlying these rapid decisions are largely unknown, especially amongst those with extensive weapons experience and expertise. In this paper, we investigate differences in weapons experts and non-experts during an incoming gunfire detection task. Specifically, we analyzed the electroencephalography (EEG) of eleven expert marksmen/soldiers and eleven non-experts while they listened to an audio scene consisting of a sequence of incoming and non-incoming gunfire events. Subjects were tasked with identifying each event as quickly as possible and committing their choice via a motor response. Contrary to our hypothesis, experts did not have significantly better behavioral performance or faster response time than novices. Rather, novices indicated trends of better behavioral performance than experts. These group differences were more dramatic in the EEG correlates of incoming gunfire detection. Using machine learning, we found condition-discriminating EEG activity among novices showing greater magnitude and covering longer periods than those found in experts. We also compared group-level source reconstruction on the maximum discriminating neural correlates and found that each group uses different neural structures to perform the task. From condition-discriminating EEG and source localization, we found that experts perceive more categorical overlap between incoming and non-incoming gunfire. Consequently, the experts did not perform as well behaviorally as the novices. We explain these unexpected group differences as a consequence of experience with gunfire not being equivalent to expertise in recognizing incoming gunfire.

## Introduction

The “10,000 hours rule” is a popular claim that expertise develops over the course of a cumulative 10,000 hours of practice at a particular task. The implication from such a statement is that experience is concomitant with expertise, though one need not imply the other. Such a claim originated from a study showing that musicians of a high-performing caliber all had practiced a cumulative 10,000 mean hours in their lifetime [1]. But as the legendary professional football coach, Vince Lombardi, famously said, “Practice doesn’t make perfect. Perfect practice makes perfect.” Furthermore, the extension of the 10,000-hour rule to other domains of expertise is more tenuous, though the reason for the breakdown of this extrapolation remains unclear.

More rigorous assessments of the role of expertise in decision-making have focused on task-specific advantages that highly-trained individuals exhibit when compared to control subjects. For instance, decision-making experiments in musical contexts have revealed both behavioral and neural markers of musical expertise in musicians [2,3]. In another instance, decision-making experiments in sports contexts have revealed comparable markers on both behavioral and neural metrics for highly-trained athletes [4,5].

Individuals that are highly trained to perform in certain environments also exhibit measurable advantages over those who regularly spectate such environments. For instance, basketball players showed both behavioral and neural differences from avid basketball-watching fans when watching video of basketball playing [6]. The implication from such a study is that both stimulus experience *and* task-related, specifically action-related, expertise are vital factors in grading decision-making domain expertise. But, the extent to which experience implies the decision-making expertise or vice versa remains unclear.

In this study, we sought to tease apart the difference between experience and expertise. The stimulus domain we studied is not accessible to a large percentage of the general population and so it provided an opportunity to recruit control subjects with little or no experience, let alone task-relevant expertise, in responding to such stimuli. Specifically, we chose to study the markers of experience and task-relevant expertise in individuals who are highly trained in the use of small arms weapons (i.e., weapons experts). In a previous study [7], we had shown that experts in small arms weapons were able to distinguish the sounds of incoming from outgoing weapons fire with high accuracy in a decision making task. Further, we showed that the neural metrics closely tracked the listeners’ behavioral performance. The present study builds upon that study [7] by taking advantage of the substantial lack of experience with small arms fire in the general population, by comparing expert with novice performance. In this way, we examine the role of experience with small arms fire, and its effect on behavioral and neural performance differences between expert and novice listening groups. Not only could such differences be relevant for military assessment and training purposes, but such differences (if they exist) could impact the assessment and training of law enforcement personnel who rely on rapid physical response to small arms fire in potentially dangerous situations. Based on the findings in music and athletic expertise, we expect that expertise should drive differences between weapons experts and novices at both the behavioral and neural level. In particular, we hypothesized that weapons expertise should drive better behavioral performance, and those differences should drive complementary group differences in neural metrics.

Insofar as experience effects could be measured, we expect that in the event there are no differences between groups, we can at least measure experience-related differences on both a behavioral and neural level, since novices are not regularly exposed to the sounds of gunfire. Specifically, we hypothesized that behavioral and neural metrics would grade experience with the selected stimulus domain, particularly showing that the experience of weapons experts would generate a measurable difference against control subjects.

The advantage of showing whether expertise and/or experience is measurable in this stimulus domain (or any other) is that not only could one identify these traits with quantified metrics, but one could also devise more focused training regimens to develop task-relevant expertise. So while neuroscientific questions can be answered with this study, there is necessarily a strong application focus implicit in considering neural response during decisions concerning gunfire.

## Materials and Methods

### Subjects

There were two groups of eleven subjects (N = 22 total), separated with regards to small arms experience (see Table 1) into an expert group (labeled E1-E11) and a novice group (labeled N1-N11). All subjects reported normal hearing and no history of neurological problems. The age differences between the groups were not significant (experts, 34.6±9.0 years; novices, 34.0±2.0 years; *t*(20) = 0.16, *p* = 0.87, independent groups t-test) and both groups were gender-matched. Placement of participants in expert and novice groups was based on the following criteria: Experts must have reported at least a 3 or 4 on a 1–4 (1 = novice to 4 = expert marksman) scale of expertise for at least one small arms weapon. Novices may have reported some experience with small arms weapons, but must not have reported experience with that weapon as greater than a one (novice) on the expertise scale.

**Table 1:** Overview of expert and novice subjects by age and gender.

Experts			Novices		
Subject Identifier	Age in years	Sex	Subject Identifier	Age in years	Sex
E1	57	M	N1	34	M
E2	34	M	N2	26	M
E3	44	M	N3	27	M
E4	34	M	N4	33	M
E5	29	M	N5	31	M
E6	34	M	N6	45	F
E7	35	M	N7	29	F
E8	26	F	N8	36	M
E9	32	M	N9	39	M
E10	29	M	N10	30	M
E11	26	F	N11	44	M

The expert subjects were recruited based on the criterion that they have extensive experience using small arms fire. All expert subjects had either served in the U.S. Armed Forces or were U.S. Department of Defense civilian employees with extensive small arms use experience. The expert subjects had regularly been using firearms since 16.1±4.3 years of age (Mean±SE, henceforth) and they self-reported experience using 7.00±0.98 different small arms weapons. Among the expert subjects, 8 self-reported the highest level of expertise (4) with at least one weapon. In contrast, the novice subjects were recruited based on the criterion that they have minimal or no experience using small arms fire. The novice subjects self-reported experience using 1.18±0.42 weapons. Among the novice subjects, none self-reported a level of expertise above 1, while 6 subjects self-reported a 1 for at least one weapon and at most four weapons.

### Ethics statement

Informed written consent was obtained from all participants in accordance with the guidelines and approval of the U.S. Army Research Laboratory Institutional Review Board (USARL IRB). The USARL IRB specifically approved this study.

### Sound characteristics of small arms fire: Differentiating targets and standards

The small arms sounds used in the present study were taken from the same set of recordings used in a previous investigation of listener perception and neural discrimination of small arms events, and the reader is directed to that paper for a more thorough description of the physical parameters of the stimuli [7]. For the purposes of the present paper, we restrict our discussion of the stimuli to a basic description of the physical parameters. In brief, the sounds of small arms fire are the result of two events: 1) an explosive release of the buildup of pressure that propels a bullet from the weapon’s muzzle and 2) if supersonic (as is true for the majority of small arms infantry rifles), an acoustic shockwave, or ballistic crack produced by traveling bullet. The muzzle blast propagates roughly spherically from the weapon’s muzzle and has total duration of approximately 3–5 ms [8]. The ballistic crack propagates outward from the traveling bullet in a cone shape behind the bullet and expands away from the target line.

The ballistic crack produces a characteristic N-wave shape in the acoustic waveform (peak pressure extremes correspond to the bow and stern of the traveling bullet) that has an extremely brief rise time (1–2 μs) and brief total duration (200–300 μs).

In the Supporting Information (S1 Fig.), we show example waveforms recorded for three-round bursts of an M4 carbine measured from two relative angles to the shooter target line of fire. The top panel depicts the waveform of a fired carbine measured at a position directly along the target line and the bottom panel depicts the waveform of the same weapon being fired and measured at a position 90° to the left of the target line. Both panels show the three-round bursts waveforms, each of which are created by subsonic pressure waves emitted from the M4 barrel upon muzzle blast. The difference in the panels is in the upper panel’s ballistic crack and first reflection, both of which precede the timing of the muzzle blast. This temporal sequence of waveforms is due to the earlier waveforms being created by the supersonic movement of the bullet. The sound propagation of the ballistic crack occurs within a critical angle forward of the muzzle that is approximately 60° to the left and right of the target line [9]. Within the bounds of this critical angle, the ballistic crack is audible and, beyond this angle, the ballistic crack is not audible. Finally, the distance from the muzzle determines the relative timing between the ballistic crack and the muzzle blast. The timing differences shown in S1 Fig. (∼30ms from ballistic crack to muzzle blast) are the result of listener positions 16m from the muzzle.

Based on these basic physical relationships, we can define 2 distinct listener positions: (1) A forward-fire position, where there is a ballistic crack followed by a muzzle blast, and (2) an off-axis position, where there is only a muzzle blast. Functionally, these two basic distinctions between listening positions can be indicative of relative safety. Specifically, for actual small arms fire, the two positions demarcate whether the listener is being shot at or not and can be indicative of friendly versus enemy fire.

### Stimuli overview and behavioral paradigm

The basic audio stimulus set consisted of the recorded sounds (24 bit, 96 kHz) of an M4 carbine being fired at a U.S. Army small arms research facility at Aberdeen Proving Ground, MD. Recordings were made at microphone locations 16m directly in front of the shooter (0° relative to the shooter target line) and 16m perpendicular to the shooter (90° relative to the shooter target line). Four separate recordings of 3-round bursts of fire were recorded at both of the microphone positions simultaneously. For the 0° stimuli, the average peak level of the ballistic crack was 149.4±0.3dB, and the average time between ballistic cracks and muzzle blasts was 20.3±0.3ms. Across both the 0° and 90° stimuli the average peak level of the muzzle blast was 149.3±0.3dB, and the average time between muzzle blasts was 75.0± 0.5ms. In addition to the firing events, three 120s long recordings were made of the ambient background at the small arms range and the average level across the background was 71.0±0.2dBA. The actual audio levels presented to listeners were much quieter than these measured levels due to power constraints of playback speakers. Also, the playback level was such that the continuous noise levels never exceeded 85 dB and peak levels never exceeded 110 dB. Importantly for the target discrimination task, the average peak level of the firing sounds was greater than 78 dB above the average background noise level, and thus there was a signal to noise ratio of greater than 2:1 in each trial.

All stimuli were down-sampled to 16-bit, 44.1 kHz for experimental playback to subjects. Using a digital audio workstation (Logic Express 9.0, Cupertino, CA), 27 unique audio scenes were created by mixing the 8 unique firing events and 3 unique backgrounds with replacement. Each audio scene had at minimum of 21 and at maximum 26 small arms 3-round bursts of fire. Two categories of firing events were used in each block, shots from 0° (targets) and 90° (standards). The order of shots for each of the 27 sequences was the same as that used in in an earlier publication [7], and that paper includes a key identifying the specific ordering of 0° and 90° shots for each of the sequences, as well as a sample audio scene as Supplemental Material. The target sounds in the sequence were always the 0° shots and occurred less frequently (23%) than the 90° standard shots (77%). The firing events occurred on a jittered inter-stimulus interval (ISI) of 3104±43ms. There were a total of 663 stimulus events (513 standards, 150 targets) across the 27 unique audio scenes presented to subjects. The ordering of targets and standards in a given sequence was randomly determined, and the average number of standards preceding a target in a sequence was 3. The order of sequences presented to subjects was randomized and no scene was heard twice.

**Fig 1:**
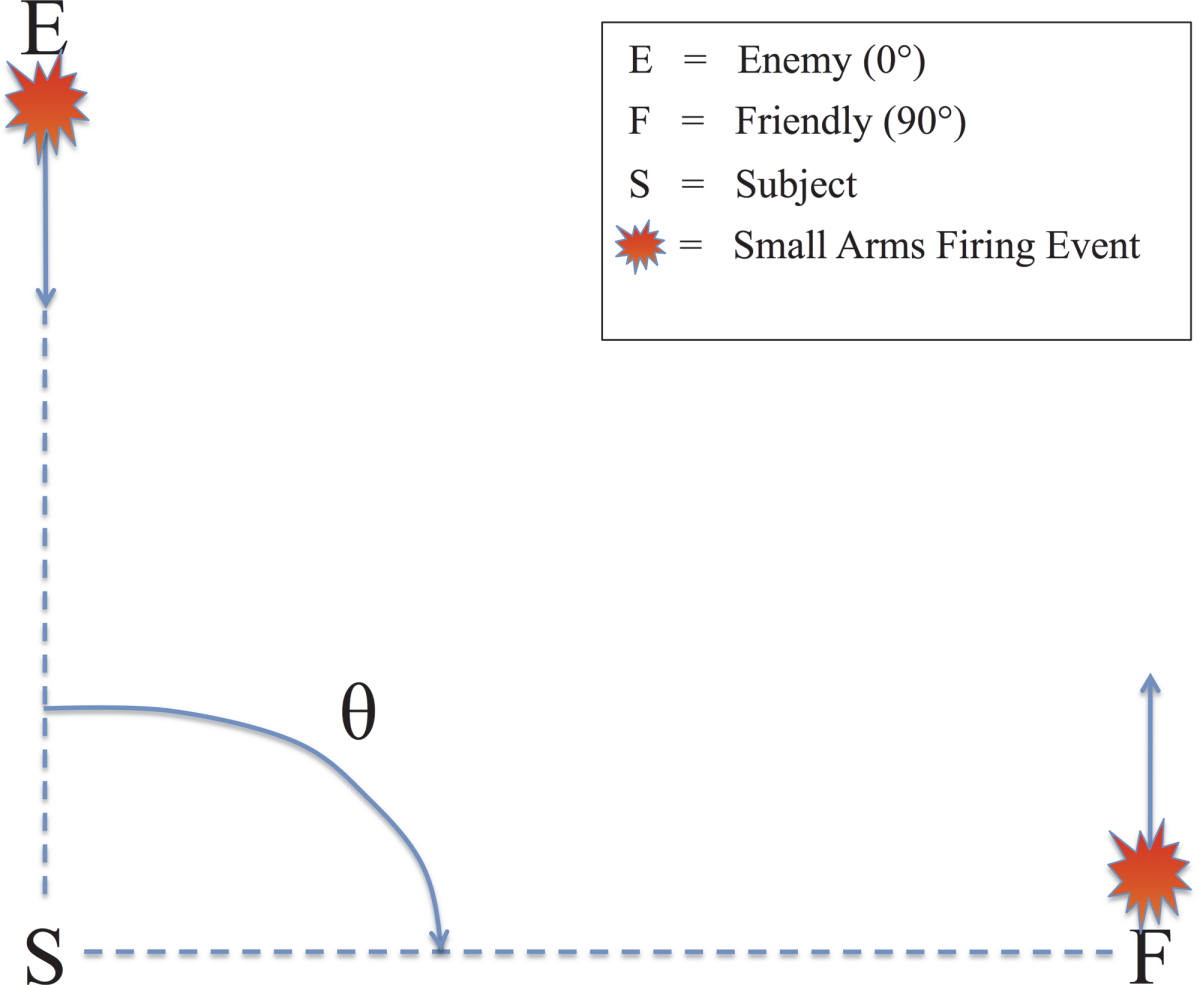
Schematic of the simulated small arms fire localization task. θ denotes the angle of incidence between the subject (S) and the firing event as conveyed by audio recording [7]. **Firing events from θ = 0° were labeled “friendly” (F) and θ = 90° were labeled “enemy” (E). Subjects were instructed to identify the angle of incidence of the firing event from these two possible choices as quickly as possible.**

All audio stimuli were presented on a Dell A525 speaker system that included 2 satellite speakers placed to the left and right, directly in front of the subjects (12” away), and a powered subwoofer placed on the floor in front of the listeners. There was no level panning to provide spatial information about the relative angle of the firing event. Rather, subjects could only determine the relative direction of the firing events based on non-spatial cues, specifically the presence or absence of the ballistic crack in the sound recording. Fig. 1 shows the scene described to the subjects in which they were making decisions, with an enemy shooting up-range at an observer at a relative angle of 0° (forward-fire position) and a friendly shooting down-range from the observer at a relative angle of 90° (off-axis fire). Therefore, a button response to 0° was labeled ‘shoot back’ and to 90° ‘all OK’ to provide the subjects a more realistic experimental context. A Dell Precision 530 Workstation was used to present the audio stimuli using E-Prime 2.0 (Sharpsburg, PA). The subjects sat in a sound-attenuated room with their eyes closed to minimize eye-blink artifacts. Despite increased alpha power, this technique has been used extensively in auditory perception tasks with EEG, mitigating any potential concerns of overpowering the ERP [10,11].

The subjects were instructed to identify the relative angle of the firing event as quickly as possible using the 1 and 2 keys on a computer keyboard. All button responses were executed with the right hand index and middle finger, regardless of handedness. Subjects received practice with the procedure and basic stimuli before beginning the experimental session by listening to and making responses to example sequences, with feedback provided. When an accuracy of 80% was reached, the experimental blocks began and EEG data were recorded. During the EEG recording, the subjects received no feedback on their performance.

### Electroencephalography data acquisition

The start of each 3-round burst of fire was the stimulus event by which EEG locking occurred. Stimulus events were passed to the EEG recording through a TTL pulse in the event channel. Stimulus events produced by E-Prime provided a point of reference then for response latencies. In post-hoc analysis, response events were added to the EEG via their latencies from the stimulus event.

EEG data were acquired using a BioSemi Active Two AD Box ADC-12 (BioSemi, The Netherlands) amplifier from 64 active scalp electrodes. All channels were referenced to BioSemi’s CMS/DRL electrodes made for use with the Active Two box. Data were sampled at 2048 Hz.

### Behavioral data analysis

We analyzed behavioral performance by examining differences between (expertise: expert versus novice) and within groups (stimulus: 0° versus 90°) using separate 2 X 2 mixed-model Analyses of Variance (ANOVAs) for the dependent measures of subject identification accuracy and reaction time.

### Neural data analysis: Event-related potentials

A software-based 0.5Hz high-pass filter was used to remove DC drifts and 60Hz and 120Hz (harmonic) notch filters were applied to minimize line noise artifacts. These filters were designed to be linear-phase to minimize delay distortions. Specifically, 2^nd^ order Butterworth filters for both the notch filters and the high-pass were combined and made into a finite impulse response (FIR) filter.

In stimulus-locked epoching (-1000ms to 1000ms), the average pre-stimulus baseline was removed (-1000ms to 0ms). After epoching to stimulus events, an automatic artifact epoch rejection algorithm from EEGLAB [12] was run to remove all epochs that exceeded a probability threshold of 5 standard deviations from the average amplitude (autorej.m, startprob = 5, maxrej = 100). Across all subjects, epoch rejection left 96.5±2.5 TC and 420.3±16.8 SC trials.

Similarly, in response-locked epoching (-1500ms to 500ms), the average baseline was removed from 1000ms before the stimulus and the same automatic artifact epoch rejection algorithm was run. The post-response data is kept to observe any potential post-evaluative neural response, such as that shown in the error-related negativity (ERN) [13].

We performed a traditional event-related potential (ERP) analysis of the filtered, epoched and artifact-removed EEG to discriminate neural response based on correct localization (e.g., [2,14]). Only responses within 1000ms of the stimulus onset were considered correct. We chose this threshold because preceding analyses that employ overt responses and neural data discrimination do not consider trials that exceed this time after the stimulus [10,15,16]. Also, this threshold allowed us to more directly compare behavioral and neural results across similar experiments (e.g., the auditory oddball paradigm used in [10]). All correct identifications of 0° events were labeled target correct (TC) and those of 90° events were labeled standard correct (SC).

### Neural data analysis: Single-trial discrimination

Due to the need for *a priori* electrode and time selection in the analysis of ERP differences, we performed a single-trial analysis of the same data used in the ERP analysis. We chose an exploratory method of data analysis particularly to avoid *a priori* electrode and time selection for our EEG data analysis. Logistic regression was used as a classifier to find an optimal projection for discriminating between TC and SC over a given time window [17–19]. Specifically, we defined a training window starting at either a pre-stimulus or post-stimulus onset time τ, with a duration of δ, and used logistic regression to estimate a spatial weighting vector ${\vec{w}}_{\tau ,\delta }^{T}$ that maximally discriminates between EEG sensor array signals *X* for each class:

$$\vec{y}={\vec{w}}_{\tau ,\delta }X$$ (1)

*X* is an *N x T* matrix (*N* sensors and *T* time samples). The result is a ‘discriminating component’ $\vec{y}$ that is specific to activity correlated with each condition, while minimizing activity correlated with both task conditions. The term ‘component’ is used instead of ‘source’ to make it clear that this is a projection of all activity correlated with the underlying source. For our experiments, the duration of the training window (*δ*) was 50ms and the window onset time (*τ*) was varied across time in 25ms steps, covering (0, 1000)ms for stimulus-locked epochs and (-1000, 1000)ms for response-locked epochs. We used the re-weighted least squares algorithm to learn the optimal discriminating spatial weighting vector ${\vec{w}}_{\tau ,\delta }^{T}$ [20]. Since our classifier (**w**) is an optimal projection in electrode space, the separation between TC and SC data incorporates all electrodes at once, rather than an *a priori* selection of electrodes of interest. Furthermore, because of our correction for multiple comparisons in time (described below), the classifiers learned at each time window are graded by significance in terms of how well they discriminate TC from SC EEG data. Thus, no *a priori* selection of time points is necessary either. In this way, we proceeded to examine the differences in the EEG response on the group level.

After solving for optimal discriminating spatial vectors in each window, we computed the electrical coupling coefficients between the component and its underlying sensor data, as shown in [18]:

$$\vec{a}=\frac{X\vec{y}}{\vec{y}\cdot \vec{y}}$$ (2)

These coupling coefficients quantify the correlation between the discriminating component and the underlying sensor data. This calculation—also called the ‘forward model’—provides a functional neuroanatomical interpretation of the resultant discriminating activity because it is calculated for each of the N sensors. The forward model, $\vec{\alpha }$, thus allows a topological representation of how strongly each electrode discriminates for one condition versus another. We used these forward models as an analog to traditional ERP topological plots, where now condition-discriminating activity is plotted at each electrode, rather than merely greater or lesser voltage.

We quantified the performance of the linear discriminator at each time window by the area under the receiver operator characteristic (ROC) curve. The ROC curve measures the rate of false positives vs. true positives and we calculated it using a leave-one-out approach [21]. Specifically, we trained the discriminator on all trials but one and then tested its classification ability, thus making each point on the ROC curve with a newly left-out trial. The area under this curve is maximal (1) for a perfect discriminator and chance (0.5) for a poor one. We refer to the area under the ROC as *A*_*z*_.

We quantified the statistical significance of *A*_*z*_ in each window (*τ*) by a relabeling procedure. This was done because it is possible to obtain discrimination above chance (0.5) just by virtue of the A_z_ calculation being done at multiple windows (i.e., there is a multiple comparisons problem in time that needs correction). Therefore, to grade statistical significance of each A_z_, we randomized the truth labels for sensor data of each class and retrained the classifier, generating a null distribution of A_z_ values and therefore a corresponding *p*-value at each window.

We utilized these null distributions in two ways, first as a means for within-subject analysis and second for between-subject (i.e., group-level) analysis. For within-subject analysis, we assessed subject-level significant discrimination using the false discovery rate (FDR) at a *p* = 0.05 threshold [22]. In particular, we applied a FDR correction for each *p*-value obtained from the null A_z_ distribution of a particular window. In this way, we calculated the number of significant (discriminating) windows for each subject. This was done for each locking condition as well. In group-level analysis, we averaged the A_z_ vs. time curves of each subject to create a group-level A_z_ vs. time curve. To create a group-level null A_z_ distribution at each window, we combined the distributions of each averaged subject, thus creating a *p*-value for the group-level A_z_. We could have once again used the more sensitive FDR correction on the *p*-values of each window, but we instead chose to use the more stringent Bonferroni correction because of the greater resolution of the null A_z_ distribution.

The number of permutations for each locking condition varied because of the different number of multiple comparisons in time. Since response-locked epochs contained twice as many windows as stimulus-locked, we calculated fewer permutations at each response-locked window due to computational constraint, though a suitable number for both subject- and group-level analyses. In particular, for the stimulus-locked analysis, we calculated A_z_ values for 250 label permutations at each of the 39 windows of each subject. For response-locked analysis, we performed 50 such permutations at each of the 79 windows of each subject. These numbers of permutations provided a suitable null distribution to evaluate within-subject discrimination using the FDR correction (*p* = 0.05) in both locking conditions. For group-level analysis, we utilized the 11x79x50 = 43450 permutations at each response-locked window and the 11x39x250 = 107,250 permutations at each stimulus-locked to estimate the null distribution of group-level A_z_ values. All group-level results are thus reported at *p* = 0.05, with a Bonferroni correction applied.

Finally, we also examined the single-trial variability of the discriminating component. The single-trial variability, here called the *EEG image*, is the time course of the discriminating component (**y**) in each epoch and it is generated by applying the classifier (${\vec{w}}_{\tau ,\delta }^{T}$) to the sensor data (*X(t)*) for a given value of τ [23]. Here, we used the τ for which A_z_ was maximum because by definition it shows on a trial-by-trial basis when maximum discriminating activity occurs at each window. We thus used the EEG image to more closely examine the relationship between discriminating neural activity and the button-response on a single-trial basis.

### Neural data analysis: Source localization

To complement the forward model analysis, we used the training window of optimum discrimination in epoch-time to inform a source localization analysis. Specifically, we used the classification results of behaviorally correct trials (i.e., TC and SC). We selected the window at which the *A*_*z*_ value was maximum for each subject. Using this marker in time, we trial-averaged the sensor data across all epochs that were either TC or SC and time-averaged within the selected window, creating a grand average ERP for each subject and for each firing event angle at this time of peak classifier discrimination. We then utilized a source localization algorithm (sLORETA [24]) to calculate the most likely cortical source distributions for each ERP. These distributions were later used for hypothesis tests between stimulus conditions (within group) and within stimulus conditions (between groups). For hypothesis tests within a group, we used a paired t-test to compare the mean source distributions at each voxel. For hypothesis testing between groups, we used an independent groups t-test on the log of the ratio of averages, which is similar to the f-ratio, as that statistic captures whether variance within the groups is different than between the groups. For multiple comparison correction in source space, we used the statistical non-parametric mapping (SnPM) method built into sLORETA [25,26]. All parameter choices are shown in S1 Table in Supporting Information.

### Statistical hypothesis testing of differences between experts and novices

We used the preceding behavioral, single-trial and source localization analyses to drive statistical hypothesis testing on group-level differences between experts and novices. In particular, we performed hypothesis tests on total accuracy, condition-specific accuracy, response times, the number of significant discrimination windows, the values and times of maximum A_z_, the strength of the discriminating component preceding each button-response, and cortical source activity. We also tested the relationship between neural and behavioral metrics with Pearson correlation. In particular, we examined the relationship between the number of significant discrimination windows and accuracy.

## Results

### Behavioral performance

We found trends indicating better behavioral performance among the novices and summarize these findings in Table 2.

**Table 2:** Summary of group behavioral performance metrics.

	Accuracy (0°)	Accuracy (90°)	Response time (0°)	Response time (90°)
Experts	0.86±0.04	0.88±0.05	672±23ms	650±25ms
Novices	0.91±0.03	0.94±0.02	614±30ms	614±30ms

Accuracy: Mean±SE

Response time: Mean±SEM

Both groups exhibited fairly high levels of accuracy for all stimuli (> 86%), with novices trending towards better accuracy, but we found no significant differences between groups. We performed a split-plot ANOVA with stimulus type (0° versus 90°) as the within-subjects factor and expertise (expert versus novice) as the between-subjects factor. We found no significant main effect of stimulus type, F(1,20) = 0.81, p = 0.38. Although novices showed a mean accuracy that was higher, the main effect of expertise was not significant, F(1,20) = 1.34, p = 0.26. We also found that there was no significant interaction between expertise and stimulus type, F(1,20) = 0.01, p = 0.91.

We also performed an ANOVA on response time data using the same between-subject and within-subject factors. We found no main effect of stimulus type, F(1,20) = 1.69, p, 0.21. Despite novices having a mean response time that was shorter than experts, we found no significant difference between the groups; F(1,20) = 1.69, p, 0.21. Also, we found no interaction between stimulus type and expertise. Interestingly, novices showed no difference in mean response time for either stimulus, whereas experts did, though these are only trends and not statistically significant differences.

### Neural markers of correctly identified firing localization: Event-related potential analysis

**Fig 2:**
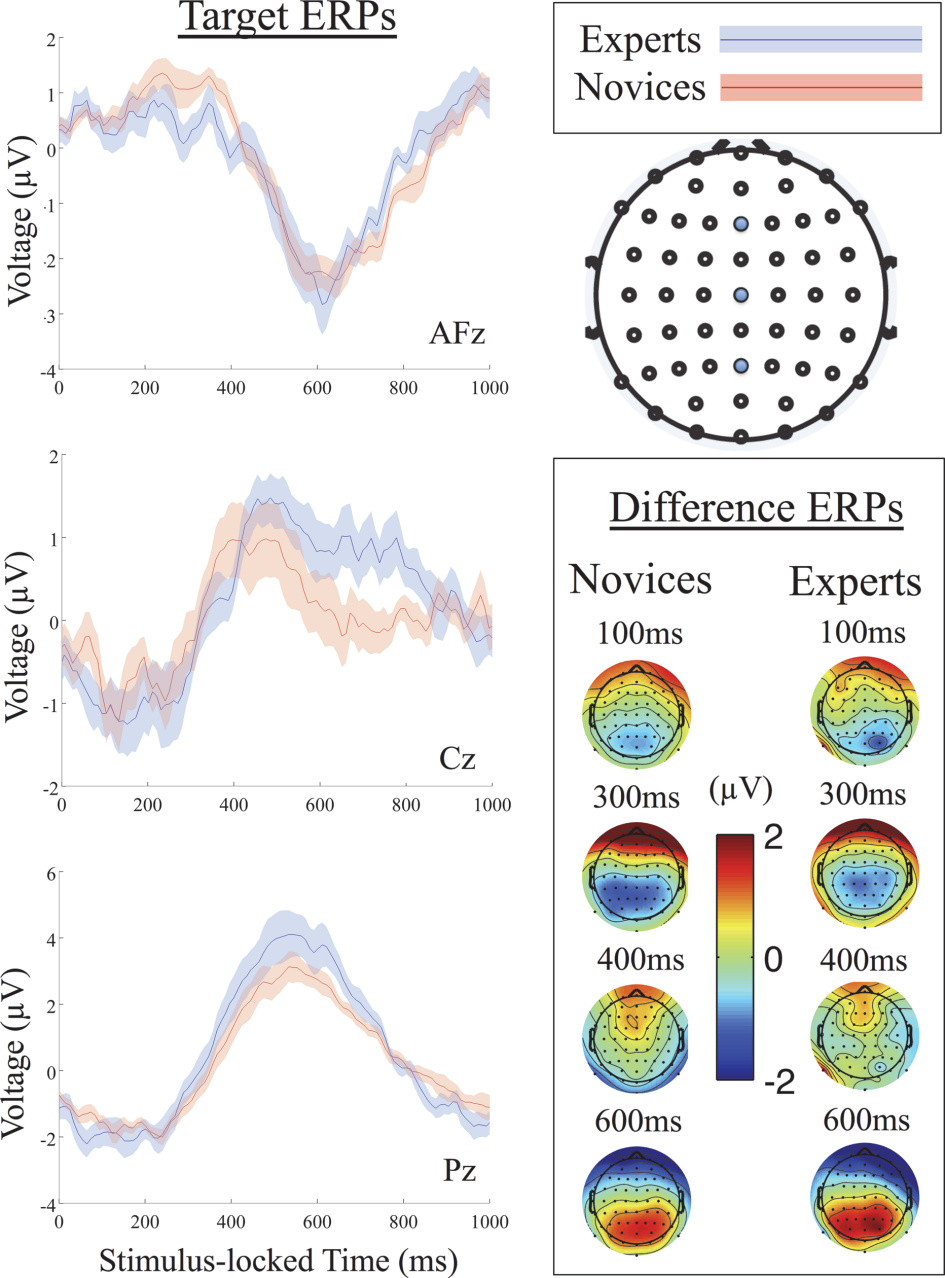
Group-level ERP analysis. The group-level mean target ERPs for three central electrodes (AFz, Cz and Pz) are shown with experts in blue and novices in red. The inset shows grand average difference ERPs for each group, with experts in the right column and novices in the left.

We investigated neural group differences using a traditional ERP analysis. Fig. 2 shows a summary of this analysis. Visual differences between experts’ and novices’ target mean ERPs are clear along standard midline electrodes (AFz, Cz and Pz), with shading showing standard error (SE) about the mean. For instance, the AFz ERP shows separation between the groups on 300–400ms and near 800ms. Cz shows a faster, though weaker, P3 response in the novices than in the experts, while showing separation between the curves on 650–850ms. Finally, Pz shows a P3 that is higher for experts than for novices.

The inset to Fig. 2 shows a visual comparison of scalp grand average difference ERPs (target—standard) at the indicated time windows ({100, 300, 400, 600}ms). Experts’ scalp plots show an asymmetry at 100ms and 400ms not seen in novices, as well as a lesser negative occipital component at 300ms and a greater positive occipital component at 600ms.

Despite these visual differences on the group level, statistical hypothesis testing of ERPs between groups suffers from a problem of multiple comparisons in electrode space and time. Furthermore, since we are using non-traditional stimuli (i.e., gunshot sound recordings), there is no previous literature we can use as a basis for electrode or time selection. Therefore, we have forgone such an *a priori* selection as done in other ERP studies. Instead, we evaluated group-level differences in neural response to either stimulus type with the results of our single-trial analysis.

### Neural markers of correctly identified firing localization: Stimulus-locked analysis

**Fig 3:**
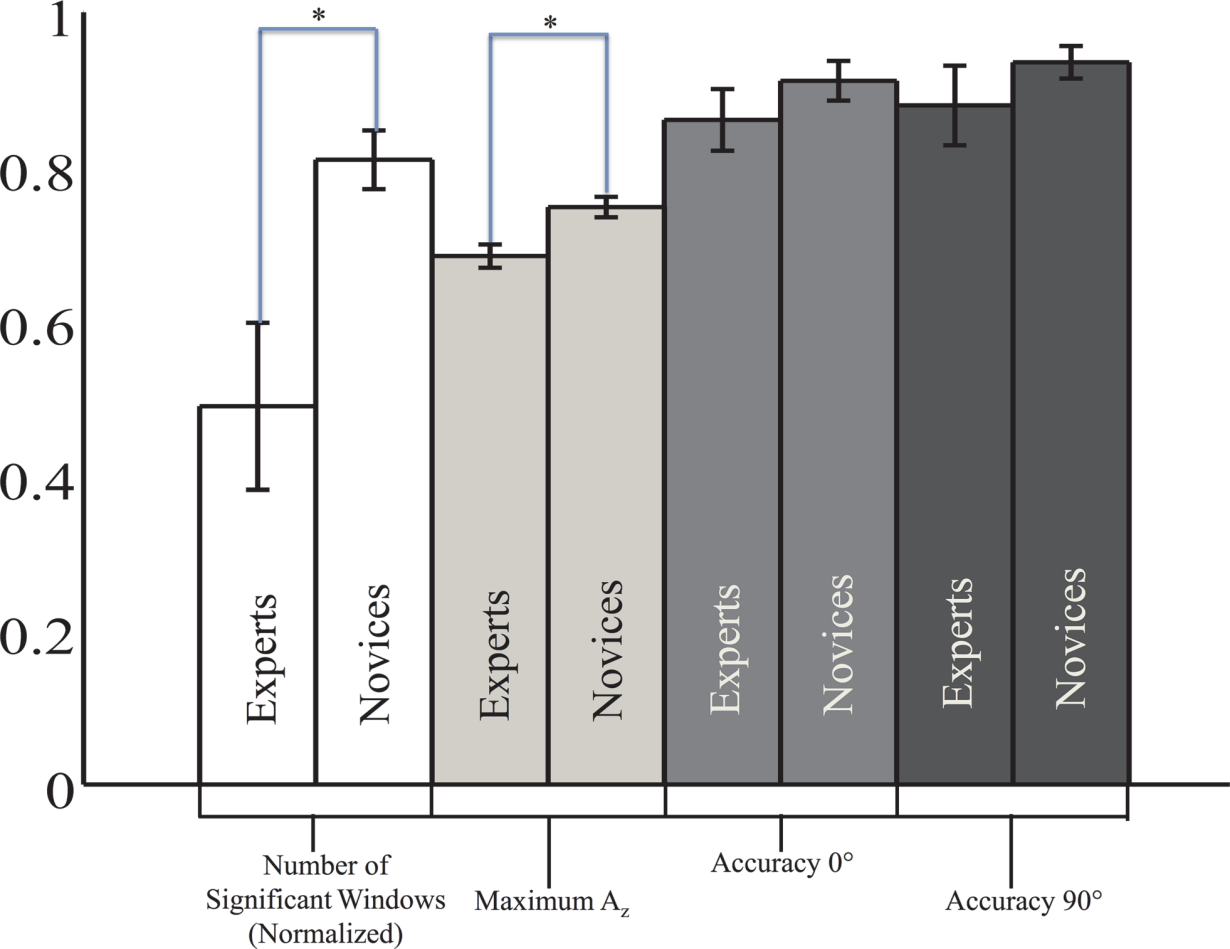
Behavioral and neural metrics for experts and novices exhibiting stimulus-locked significant discrimination. We found that the experts’ accuracy was not significantly less than that of the novices for either 0° or 90° stimuli (p > 0.28, both independent groups t-tests, |t| < 1.10, DOF = 20). However, we did find a significant difference in neural metrics at the group level, finding higher maximum values of A_z_ and more significant windows of classifier discrimination for novices, (p < 0.02, both independent groups t-tests, t < -2.78, DOF = 20).

We first examined whether there was a difference in the number of time points for which experts and novices showed significant A_z_ values. Not all small arms experts exhibited at least one time window of significant discrimination. Specifically, E1, E3 and E6, exhibit no windows of neural activity discriminating TC vs. SC conditions. In contrast, all novices exhibit at least 21 windows of significant discrimination. Comparing group-level performance using this criterion, we find that novices exhibit more windows of significant discrimination than experts, t(20) = -2.79, p = 0.01, independent groups t-test. The white bars of Fig. 3 show this group-level difference (indicated by an asterisk (*)), where we have normalized the number of significant windows by the 39 total windows. The grey bars to the right are the behavioral accuracy data for reference.

We then found further group-level differences when comparing windows of maximum classifier discrimination. Specifically, the value of maximum *A*_*z*_ for novices was higher than that of experts, t(20) = -3.17, p = 0.01, independent groups t-test, with experts showing a maximum significant *A*_*z*_ of 0.68±0.02 and novices a maximum *A*_*z*_ of 0.75±0.01. The light grey bars of Fig. 3 show this group-level difference (indicated by an asterisk (*)). Although we did not see significant differences in the near-ceiling behavioral accuracy (or the RT data), we now see significant differences in the single-trial analysis of the neural data, with a significantly stronger neural response to the stimuli by the novice listeners. In contrast to the significant differences in peak and extent of TC vs. SC classifier discrimination at the group-level, there were no significant differences in the time courses of discrimination between experts and novices; the time to maximum *A*_*z*_ was 502±129ms for experts and 509±35ms for novices, t(20) = -0.05, p = 0.95, independent groups t-test. Additionally, there was no significant difference in the time of the first window of significant discrimination t(20) = 0.73, p = 0.47, independent groups t-test.

### Relating neural markers of correctly identified firing localization to behavioral response

**Fig 4:**
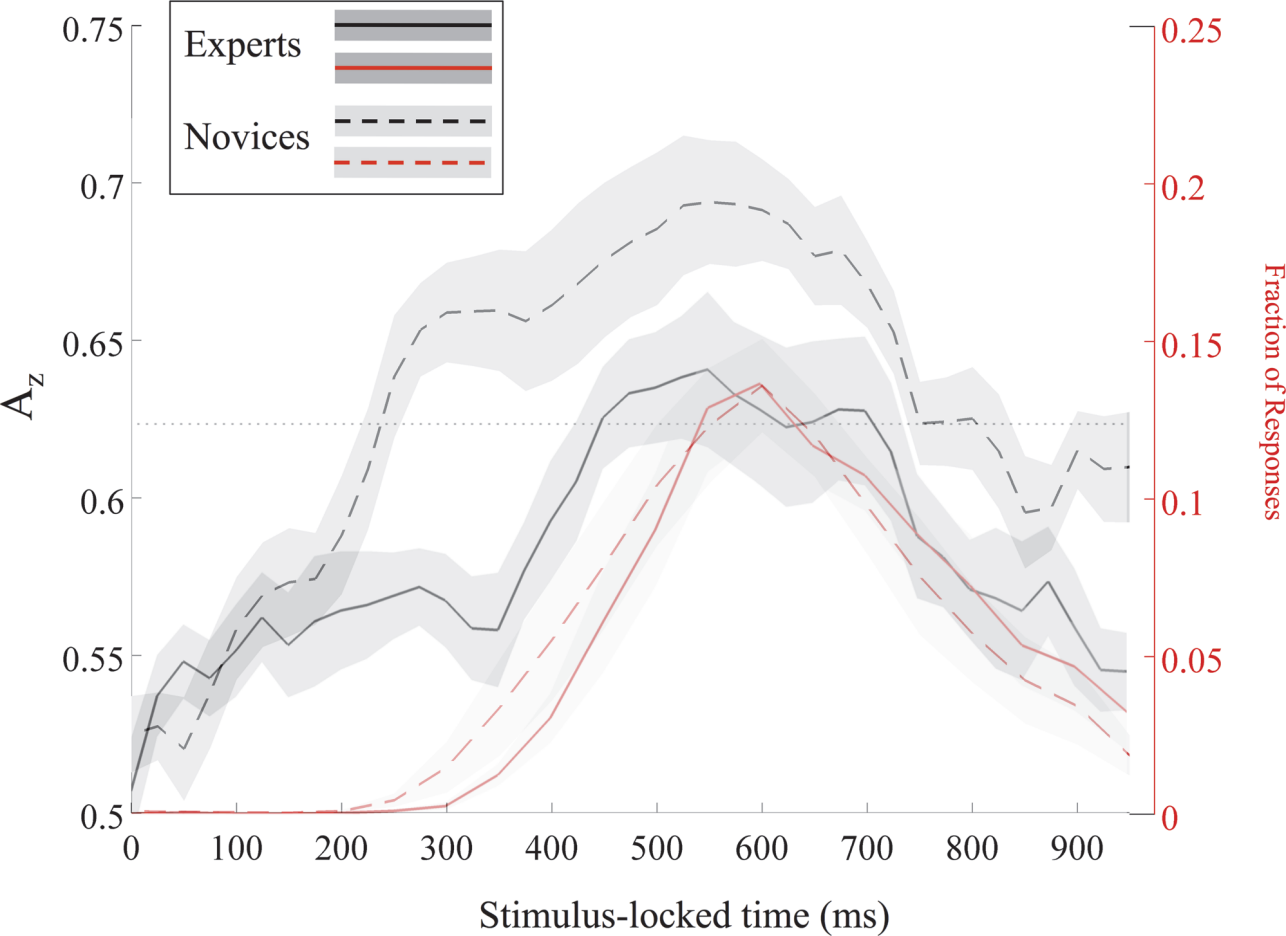
Expert and novice comparison of stimulus-locked A_z_ values (black) and normalized response distributions (red) for all stimuli. Experts’ mean values are solid lines, while novices’ are dashed. Standard error values are shaded gray for both. A_z_ values are indicated by the left abscissa in black and normalized response distribution frequency values are indicated by the right one in red. Group-level Bonferroni threshold for significant A_z_ values is shown with a dotted line.

Even though the time courses of A_z_ showed no difference at the group level, there was a significant difference between experts and novices with respect to the timing difference between neural and behavioral response. Visually observing this difference in **Fig. 4**, we aimed to uniquely quantify it with a novel metric relating neural to behavioral response. Specifically, we quantified this difference by summing the distances between the A_z_-vs.-time and behavioral response distribution curves for each subject (see mean±SEM curves for each metric in black and red, respectively, in **Fig. 4**). Looking at the data in this way, there are smaller distances between these lines for the experts than for the novices, t(20) = -2.5, p < 0.01, independent groups t-test. Consequently, we found that experts’ neural response occurs closer in time to the behavioral.

We found no consistent relationship between groups that linked behavioral performance to the neural metrics used here. In our previous study [7] we had found that there was a positive correlation for experts between the number of significant windows and behavioral accuracy for 0° (r = 0.795, p<0.01) and 90° (r = 0.812, p<0.01) stimuli. However, this correlation was either marginally or not nearly significant in the present study for novices (r = 0.55, p = 0.08 for 0° stimuli; r = 0.26, p = 0.44 for 90° stimuli).

### Single-trial variability of neural markers of correctly identified firing localization

**Fig 5:**
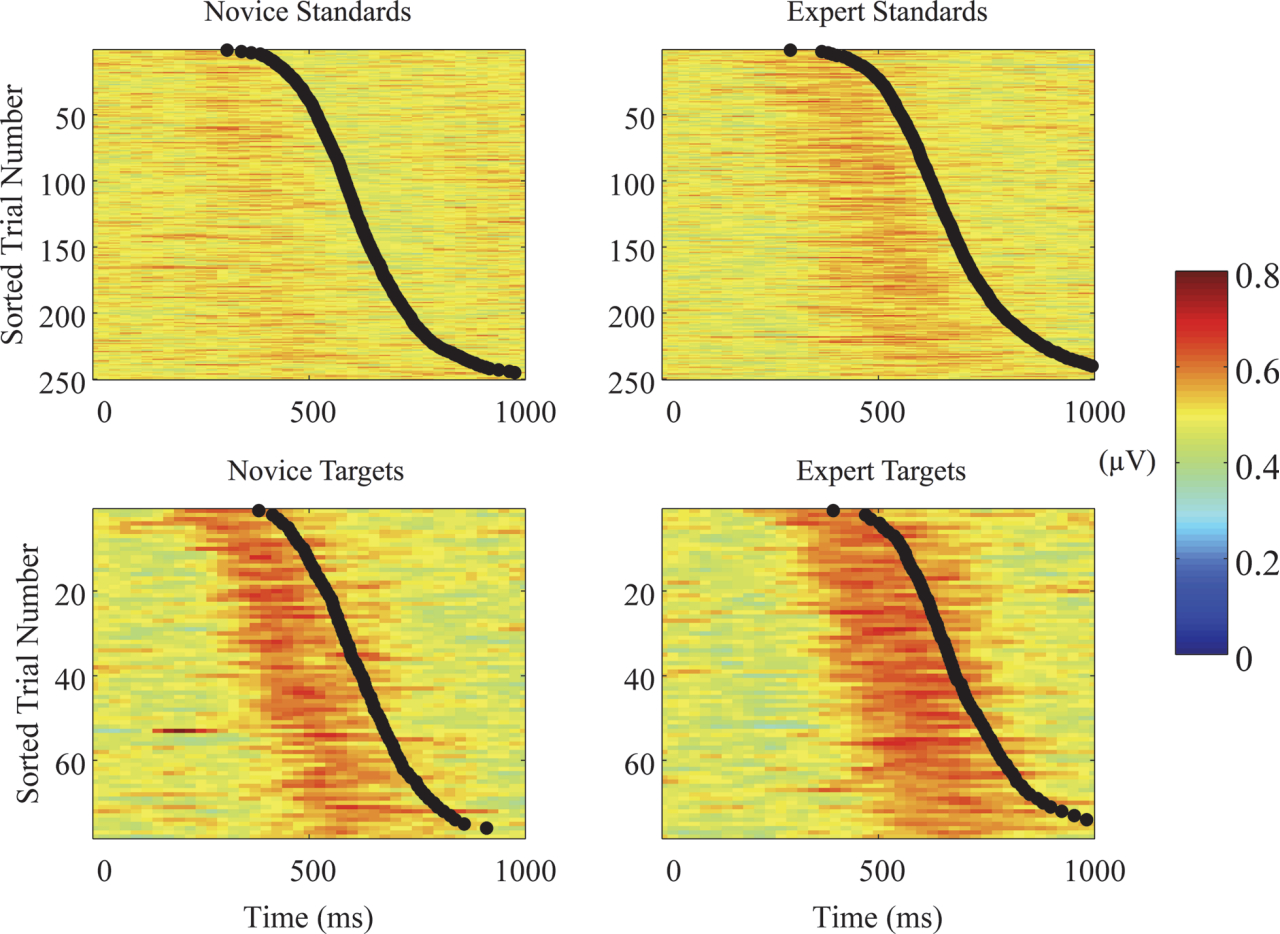
Single-trial discriminating component time course for response-time-sorted trials. A common number of target correct (TC) and standard correct (SC) trials were used across all subjects (78 targets, 250 standards). The discriminating component (y) is meaned across subjects at each sorted trial number and scaled by the logistic function.

We further examined group-level difference by examining the single-trial variability of expert and novice neural responses to each correctly identified stimulus. The EEG image averaged across subjects at each sorted trial is shown for each group and stimulus condition in Fig. 5. Both novices and experts exhibited a rise in the discriminating component (y) leading up to a button-response for target correct (TC) trials. We calculated this by finding the maximum y value for each trial, each stimulus condition and each subject, and then averaging across trials within each stimulus condition. Consistent with the significant group-level differences found for maximum A_z_, novices exhibited greater trial-by-trial maximum *y* than experts: t(20) = -2.33, p = 0.02, independent groups t-test. We calculated this by finding the maximum y value for each trial, each stimulus condition and each subject, and then averaging across trials within each stimulus condition. We also found that experts had a numerically greater mean trial-by-trial maximum y than novices for standard correct trials, but this difference was only marginally significant at a group-level: t(20) = -2.33, p = 0.07, independent groups t-test.

### Neural markers of correctly identified firing localization: Response-locked analysis

**Fig 6:**
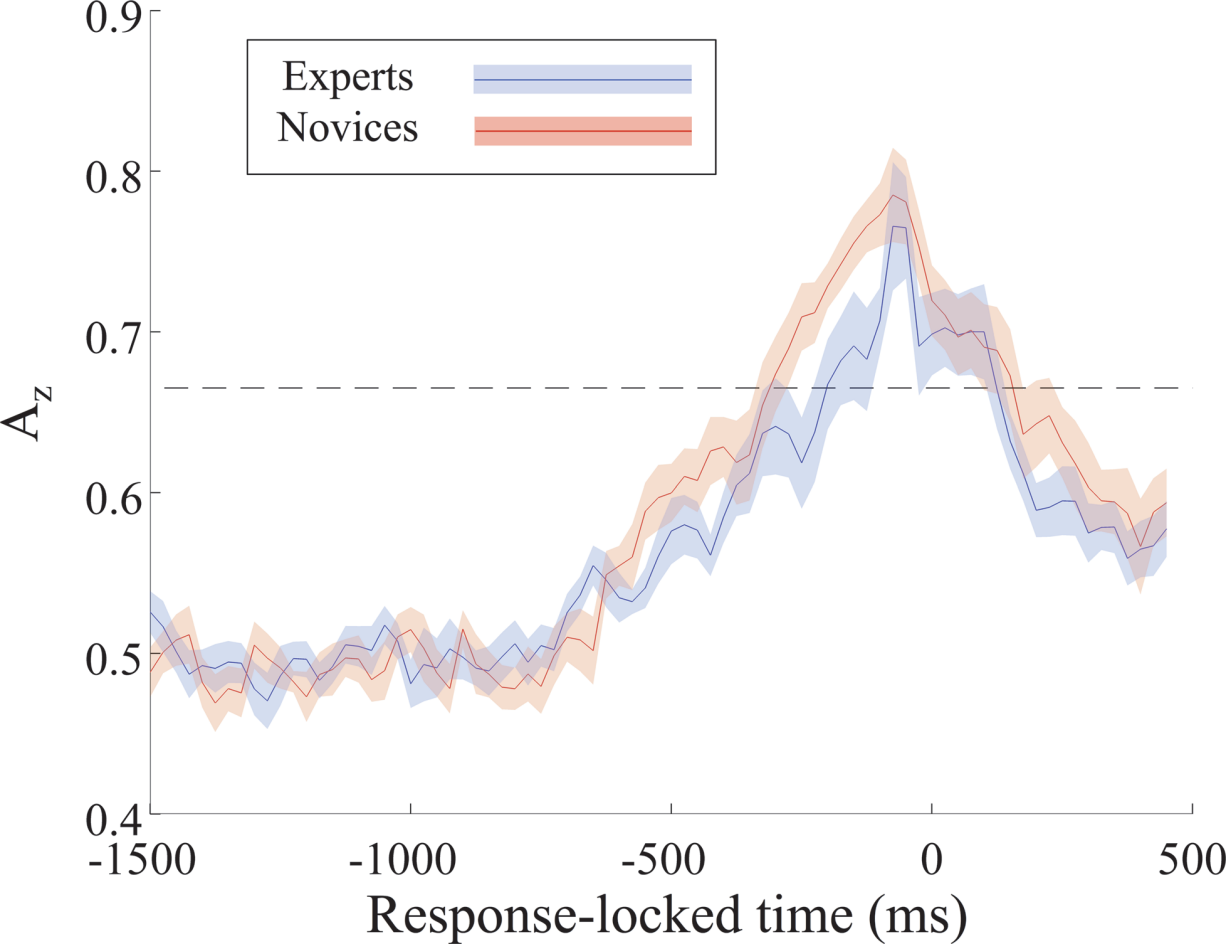
Expert and novice comparison of response-locked A_z_ values (mean in solid, standard error of the mean in shading). Group-level Bonferroni threshold for significant A_z_ values (p = 0.05) during the 1000ms surrounding the response is shown with a dashed line and extended throughout the rest of the epoch.

To further investigate group-level differences between expert and novice subjects, we also trained our classifier on EEG data locked to the response times (**Fig. 6**). Using the FDR correction across time as a measure of significance for each subject (p = 0.05), we found that novices show a greater number of windows of significant discrimination and a higher mean A_z_ value in the 1000ms surrounding the response: t(20) = -2.0, p = 0.03, independent groups t-test; t(20) = -2.46, p < 0.01, independent groups t-test. Though there is no group difference in the timing nor value of peak A_z_ (timing: t(19) = -0.15, p = 0.44, independent groups t-test; value: t(20) = -0.35, p = 0.63, independent groups t-test).

**Fig 7:**
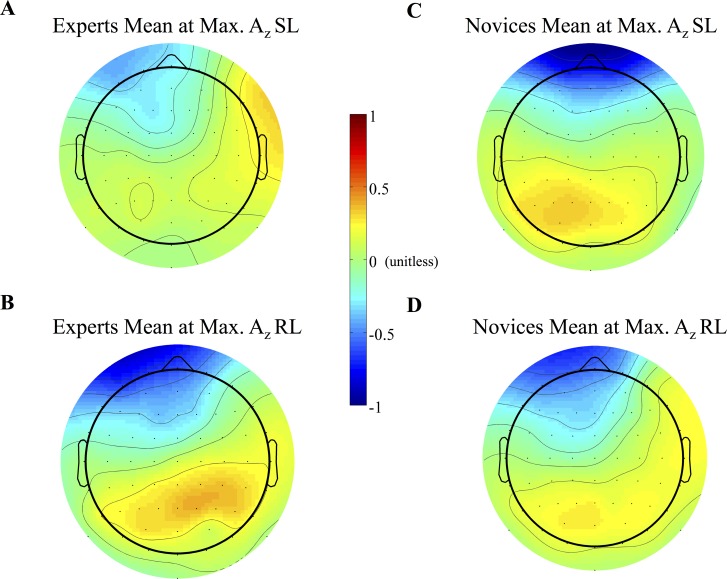
Mean normalized forward models. (A and B) Expert and (C and D) novice forward models for stimulus-locked (SL) and response-locked (RL) windows of maximum A_z_ are shown. More positive values indicate higher correlation of y with X of target correct (TC) trials, while more negative values indicate higher correlation of y with X of standard correct (SC) trials.

**Fig 8:**
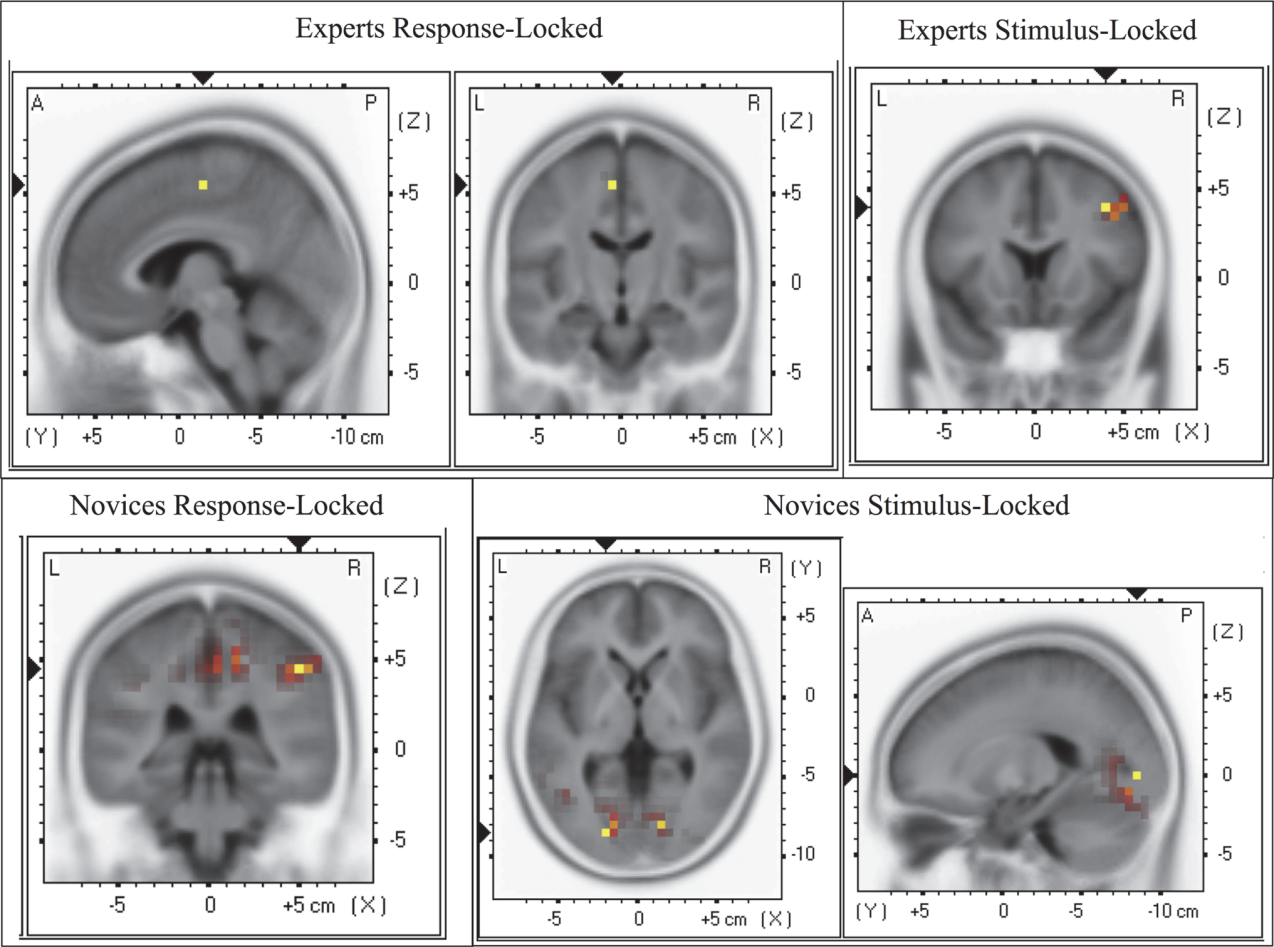
Source localization comparisons at maximum Az for experts and novices. All source activity is shown for TC > SC at a corrected threshold of p < = 0.01. Maximum Az classifiers were found in both stimulus-locked and response-locked epochs for all subjects.

**Forward and Source Models of Discriminating Activity.** We also examined the differences between stimuli and groups at the level of forward and reconstructed source models. The mean normalized forward models of each group for each locking condition are shown in **Fig. 7**. We investigated the differences that led to these plots by considering their underlying source models. We first confirmed that the differences between TC and SC trials shown on the scalp level of **Fig. 7** (orange-red values) manifested at the source level, with TC trials generating stronger current sources than SC trials. We found at the subject-specific time of maximum A_z_ that TC trials showed greater sources than SC trials within each group for both event-lockings (expert stimulus-locked: t_max_(20) = 5.87, p_min_ = 0.002, paired t-test; novice stimulus-locked: t_max_(20) = 5.98, p_min_ = 0.003, paired t-test; expert response-locked: t_max_(20) = 4.92, p_min_ = 0.01, paired t-test; novice response-locked: t_max_(20) = 7.50, p_min_ = 0.0005, paired t-test). We found that the location of these significant responses differed between groups and locking conditions (see Fig. 8), though the sensor locations for TC-discriminating activity corroborated well with the location of underlying TC-significant sources. Full list of significant responses with MNI coordinates and cortical structures are in the Supporting Information (experts: S2 Table; novices: S3 Table).

**Fig 9:**
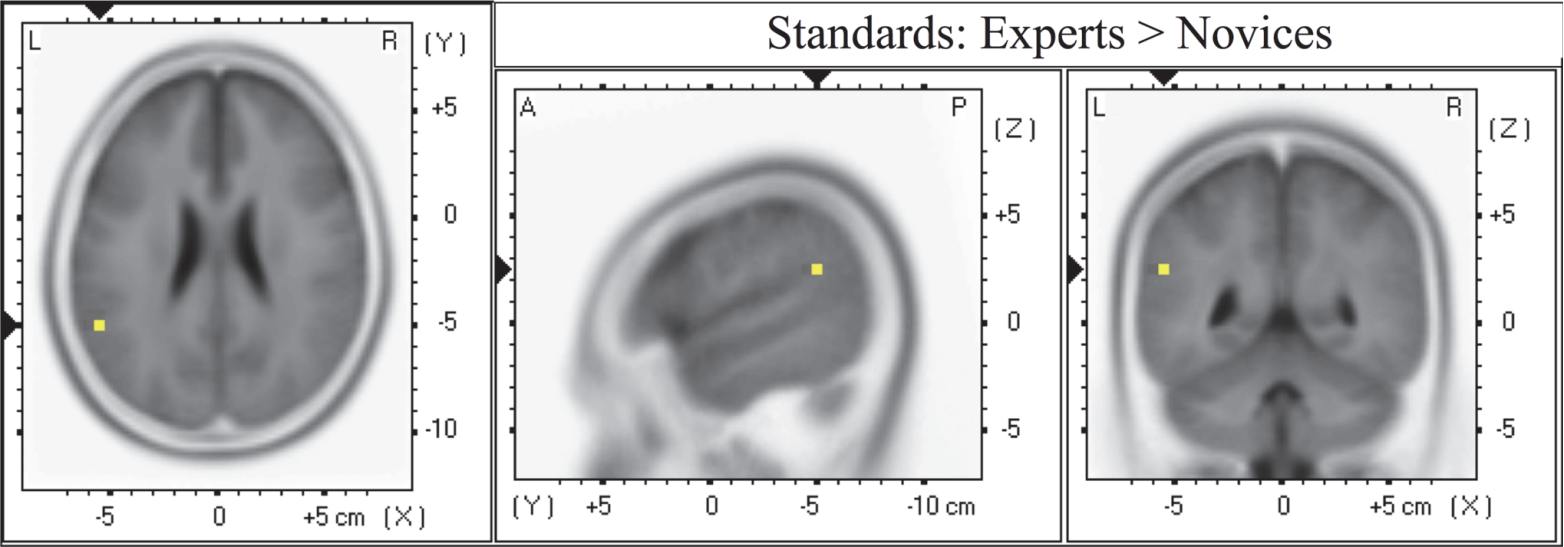
Source localization group-level comparison at maximum Az for standard correct (SC) trials. Source activity is shown for experts > novices at a corrected threshold of p < 0.05.

We also compared group-level source activity to directly test differences between experts and novices within each stimulus condition. We guided our hypothesis testing by the earlier result that experts showed a higher maximum discriminating component activity on a trial-by-trial basis in the EEG image than novices for SC trials, though this result was only significant at p = 0.07. But, when we compared the source distributions, we found that experts showed a greater current source than novices (response-locked: t_max_ = 2.35, p_min_ = 0.03, independent groups t-test; see Fig. 9 and S4 Table in the Supporting Information). No significant differences in current source were found for TC trials.

## Discussion

In this study, opposite to expectations, we have shown that weapons experts do not demonstrate a behavioral or neural advantage over weapons novices in their ability to discriminate infrequent incoming fire. Although weapons novices only demonstrated an insignificant numerical trend for a behavioral advantage over experts, these novice listeners showed several significant differences in spatial and temporal neural metrics that suggest an efficiency advantage in processing. We posit that the near-ceiling behavioral accuracy at the task studied here is a large contributor to this group-level difference being insignificant at the behavioral level, while being significant at the neural. Consequently, we suggest a significant scientific contribution of this study, beyond its immediate application to decision-making situations amidst dangerous gunfire, is that neural metrics can be sensitive to differences in task performance when behavioral differences may be masked by insensitive behavioral metrics influenced by ceiling effects.

Here, we consider our hypotheses in the context of our results and how they fit into the preexisting relevant literature. Specifically, we expected there to be clear behavioral differences between groups, with experts showing behavioral and neural advantage in processing. We found consistent numerical trends in behavioral differences, and more importantly, significant neural differences between the groups. However these results were opposite to our initial hypothesis, with novices demonstrating an advantage over experts.

### Neural difference at behavioral ceiling performance

We found that neural metrics can be sensitive to differences between groups, even when behavioral metrics are not sensitive. The lack of behavioral sensitivity can be traced to the high, ceiling performance on the task exhibited by both groups overall (> 86%). Even more telling of a ceiling effect is that in each group, 8 of 11 subjects performed at better than 90% accuracy, and at least 6 of those subjects in each group performed at better than 95% accuracy. Among novices, where behavioral accuracy was marginally higher, the ceiling effect is likely the reason why we could not find significant correlation with neural metrics, as we had done in earlier work with experts. Sensitivity problems for ceiling performance are a well-documented issue that can effectively mask important, real differences between variables (e.g. Mitchell & Jolly, 2012). In the present study, we find that even though the behavioral accuracy measures are at the high extremes of the behavioral scales, the neural metrics clearly are not, and thus remain sensitive. To our knowledge, the concurrent neuroimaging of this effect has not been studied.

In this paper, the neural differences are driven by variable experience and use-expertise of weapons experts, as compared to weapons novices. Up until the participation in the study, all experts had used 7±1 weapons and 73% of them claimed the highest level of use-expertise (4) on at least one weapon. In contrast, the novices had used 1±0 weapons and 0% claimed a level of use-expertise beyond the lowest level (1). In the case of near-peak behavioral performance seen in our results, we found that the only explanation for the neural differences in timing and strength of stimulus response originates from the use-expertise and live experience differences between the groups. Despite weapons experts and novices effecting their decisions at statistically indistinguishable times, **Fig. 4** shows that novices perceived the difference in 0° and 90° stimuli earlier than experts. We quantified this difference by noting greater separation in time between the neural discrimination line (A_z_) and the response distribution line for novices. Specifically considering correct decisions on 90° stimuli (i.e., SC trials), Fig. 5 shows that experts demonstrated a marginally stronger neural response *as if* for 0° stimuli (i.e., TC trials) immediately preceding their behavioral response on SC trials.

The source localization results within groups and between stimuli showed us that there are different neural mechanisms at play in these groups, potentially driving these observed differences in neural discrimination (Fig. 8). The experts showed strongest source differences in response-locked epochs in the supplementary motor area (see ‘Experts Response-Locked’). Novices also showed source differences in the supplementary motor area for response-locked epochs (see ‘Novices Response-Locked’), but their stronger source is lateralized to the inferior parietal lobule. The experts showed source differences also in the inferior parietal lobule for stimulus-locked epochs (see ‘Experts Stimulus-Locked’). Finally, novices showed source differences in the occipital lobe for stimulus-locked epochs (see ‘Novices Stimulus-Locked’). The response-locked epochs allow us to judge these source differences with respect to the decision, so we can see how each group culminates in the motor decision to respond to incoming gunfire: both groups employ motor planning and executive control areas to affect the motor response in such recognition. But the difference occurs when we examine how each group discriminates the incoming gunfire stimulus from the standards. Earlier work on auditory oddball has shown that any target stimulus (here, incoming gunfire sounds) can generate occipital activity, i.e., in visual areas, which is thought to result from attention demands pulling away from the visual system [10]. For the novices, we see such source activity in stimulus-locked epochs, potentially indicating that the novices are orienting to the target stimulus as an unexpected event, i.e., as an oddball, not necessarily as an incoming gunshot *per se*. Conversely, for the experts, we see activity in the lateral prefrontal cortex, which is crucial for executive functioning [27]. This activity in the experts could reflect that this group perceives the TC trials already as a stimulus on which to make a decision. Such activity is in contrast to the novices’ attention-orienting neural response. Such a difference could explain why the experts’ neural response is closer in time to the behavioral (shown in **Fig. 4**).

We also found significant differences between groups. In our analysis of the EEG images for each group, we found a trend showing that experts had a greater trial-by-trial maximum *y* for SC trials (p = 0.07). **Fig. 4** shows how these peaks in *y* closely precede the response time, effectively showing that they are motor-response driven. Our final source localization result (Fig. 9) isolates the potential origin of this difference between the groups. Using response-locked epochs, we found that experts show more source activity for novices in Brodmann Area 40, an area associated with tasks in sentence comprehension and other audio-based semantic abilities [28–30]. By engaging neural mechanisms involved in extracting meaning from auditory stimuli, the experts could be demonstrating on a neural level that there is implicit meaning in the standard stimuli that the novices do not recognize.

We speculate that this stronger source strength may be a consequence of the nature of the expert’s experience. Although these listeners have extensive experience firing small arms, their experience is largely limited to the sounds of their own weapons, or the sounds of others firing down range. This situation is equivalent to the present study’s standard stimuli where the primary sound is from the weapon’s muzzle blast only. A listener would only hear small arms fire with a ballistic crack present if they were actually being fired upon, and even if that occurred at some point for an expert listener, it would still represent a very rare event compared with the lifetime experience with muzzle blast sounds. As such, it is possible that for our expert listeners the tendency may have been to perceive all gunshots as belonging to the category of “gunshot sounds,” with little distinction as to the presence or absence of the ballistic crack. This possibility is analogous to what one finds in the perceptual organization of speech categories. For example, in English there are only two important voicing distinctions for stop consonants, one where there is no delay between release of the stop and vocal cord vibration (e.g. /*ba*/), and one where the release of the stop precedes voicing (e.g. /*pa*/). Although, other languages such as Thai use the additional distinction of a voicing lead (vocal cord vibration precedes stop release), English speakers are not sensitive to the voicing distinction and simply categorize the sound as if the offset were zero [31]. Novice listeners however, have little experience with the sounds of small arms and should not have a well-formed perceptual category for “gunshot sounds”. Consequently, novice listeners could be expected to have listened more analytically, and may have been more sensitive to the stimulus differences between the present study’s standard (90°) and target (0°) stimuli.

### Is it expertise or is it experience we are measuring?

The glaring result from our study is that our experts were not experts at the study task. In particular, experience in a stimulus domain is not necessarily commensurate with expertise at acting in that domain. The focus of our previous study [7] was to examine the effectiveness of our neural classifiers to predict performance. That behavioral performance was high for expert listeners was not a particular problem for that goal. In the present study, we had the expectation that novice listeners would perform much poorer than our experts, but that turned out not to be the case. Instead we had very high behavioral performance for both groups that was statistically indistinguishable, except for a trend for better performance by our novice listeners. Despite the similar behavioral performance, we nevertheless measured a clear difference in these two groups’ neural response for decisions based on gunshot stimuli. With user expertise not being a viable proxy for sound discrimination expertise in this case, and sound discrimination expertise demonstrated instead by the novice weapon users, live experience with gunfire is the only remaining variable that accounts for the observed group-level differences. Therefore, we have in fact shown how to measure experience with potentially dangerous gunfire using this paradigm and analysis. The experience measured here necessarily accrues with use expertise, but it does not translate to sound discrimination expertise, at least within the benign and controlled settings of our experimental paradigm. This experience and use-expertise may still transfer to sound discrimination expertise if the context of hearing the gunfire is such that the subject is actually in immediate danger. But such an aim is beyond the scope of this auditory-only study.

### Stimulus “pop-out” effects

Our results are predicated on the use of an experimental paradigm in which there is an unequal distribution between target and standard stimuli. Many studies comparing different groups of subjects use such an unequal distribution of stimuli types (refs). Our study only differs from these in experimental approach insofar as we have utilized gunshot audio recordings for our stimuli. It deserves particular mention that the differences in expertise and/or experience tested in this study are seen due to the use of such an experimental paradigm, i.e., one in which an unequal distribution of target and standard stimuli is used. In pilot testing of this paradigm, experts in small arms fire and battle situations guided the choice of paradigm, in particular because enemy gunfire does in fact “pop-out” from the rest of the scene. This is the particular logic behind our choice of making an oddball experiment out of such gunfire sequences. While our results do not provide evidence on the hypothesis of a neural response between incoming (0°) and non-incoming (90°) gunfire, they do provide evidence on the hypothesis of a neural response between *infrequent* incoming and non-incoming gunfire. Once comparing the groups, as we’ have done in the Results, it becomes clear that the stimulus “pop-out” effects due to infrequent incoming gunfire generate different spatio-temporal brain response in expert and novice groups.

### Could alpha power be driving expert vs. novice group differences?

A valid concern from the choice of experimental paradigm is that alpha power contamination in the EEG recording could drive group differences. For instance, it is possible that, due to the eyes-closed EEG recording, expert subjects’ alpha activity is solely driving the logistic regression classification and thus the different neural response from what is seen in novices. The eyes-closed EEG recording technique for auditory experiments has been used extensively in target detection and oddball experiments [10,11]. It has also been used in auditory expertise studies involving musicians [2].

Nevertheless, we tested the hypothesis of alpha power contamination driving group differences diretly. We used the event-related spectral perturbation [12] to measure differential activity due to the target in comparison to the standard stimuli, relative to the stimulus onset. We show the mean group difference in such activity in Supporting Information (S2 Fig.) and confirm that the frequency bands from 6–30Hz show significant difference in the 1000ms post-stimulus-onset (FDR corrected for multiple comparisons in frequency band, p < 0.05). Therefore, alpha power is not the sole driver of group differences seen from differential response to target compared to standard stimuli.

## Conclusion

Contrary to our initial hypothesis, we found that weapons experts displayed trends of inferior behavioral performance to novices and that those differences translated to inferior neural performance in discrimination ability between incoming and non-incoming gunfire. Due to the novelty of gunfire sounds for novices, we found that behavioral and neural metrics graded experience with the selected stimulus domain as well. Yet, we found such experience effects among the experts too: their experience with gunfire makes the task of discriminating incoming from non-incoming gunfire more difficult. In other words, their experience with gunfire does not translate to their expertise in its auditory discrimination.

As an unexpected consequence of novices displaying trends of better behavioral and significantly better neural performance, we found that neural metrics provided a means to quantify performance difference at near-ceiling behavioral accuracy. To our knowledge, this is the first time neural metrics have been used to do so. Consequently, the neural metrics used here may provide application to soldier training and other rapid decision-making tasks in which peak behavioral performance is at a premium.

## Supporting Information

S1 Fig (Reproduced from [7]) Waveforms of gunfire recordings. Top (A): Waveform of a 3-round burst of fire for a M4 carbine recorded in 16m front of the shooter directly along the target line (0° incidence). The ballistic crack, ballistic crack reflection and muzzle blast are labeled. Bottom (B): Waveform of a 3-round burst of fire for a M4 carbine recorded 16m perpendicular to the left of the shooter target line (90°). The muzzle blast is labeled.
(TIF)

S2 Fig Event-related spectral perturbation (ERSP) of differential activity due to target compared to standard stimuli. The difference between experts’ and novices’ mean differential activity are shown (experts—novices).
(TIF)

S1 Table sLORETA parameters used for source localization comparisons. All other parameters set to FALSE besides those shown here.
(DOCX)

S2 Table Experts’ MNI coordinates in mm and cortical structures showing greater neuronal source activity for TC trials than SC trials. (DOCX)

S3 Table Novices' MNI coordinates in mm and cortical structures showing greater neuronal source activity for TC trials than SC trials. (DOCX)

S4 Table MNI coordinates in mm and cortical structure showing greater neuronal source activity for experts than novices during SC trials. (DOCX)
