# Supplementary figures and images for: Experience Does Not Equal Expertise in Recognizing Infrequent Incoming Gunfire: Neural Markers for Experience and Task Expertise at Peak Behavioral Performance
Source: PLoS One. 2015 Feb 6;10(2):e0115629. doi: 10.1371/journal.pone.0115629 (PMC4319735; doi:10.1371/journal.pone.0115629)

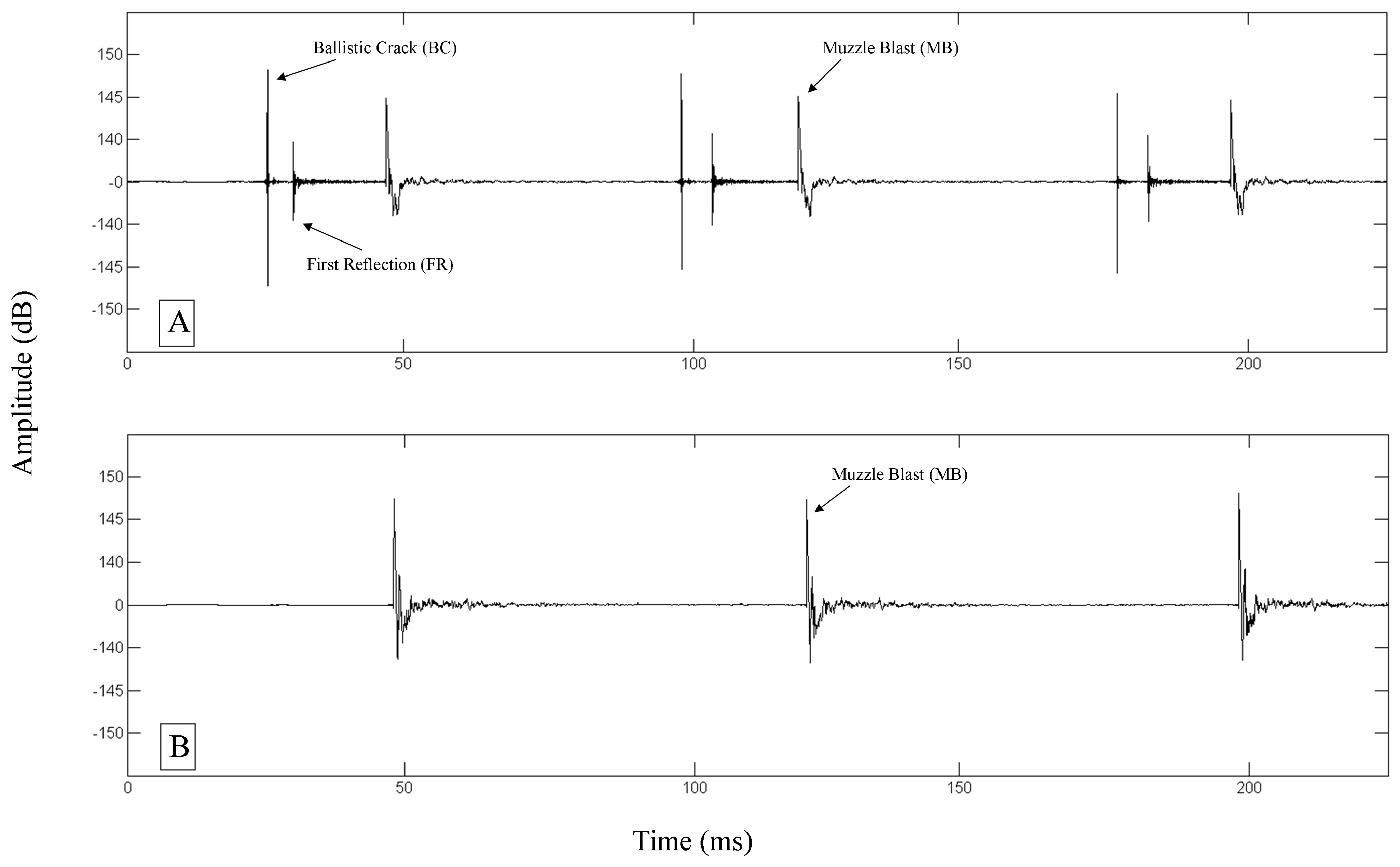

Supplement: S1 Fig — Top (A): Waveform of a 3-round burst of fire for a M4 carbine recorded in 16m front of the shooter directly along the target line (0° incidence). The ballistic crack, ballistic crack reflection and muzzle blast are labeled. Bottom (B): Waveform of a 3-round burst of fire for a M4 carbine recorded 16m perpendicular to the left of the shooter target line (90°). The muzzle blast is labeled. (TIF) [file pone.0115629.s001.tif]

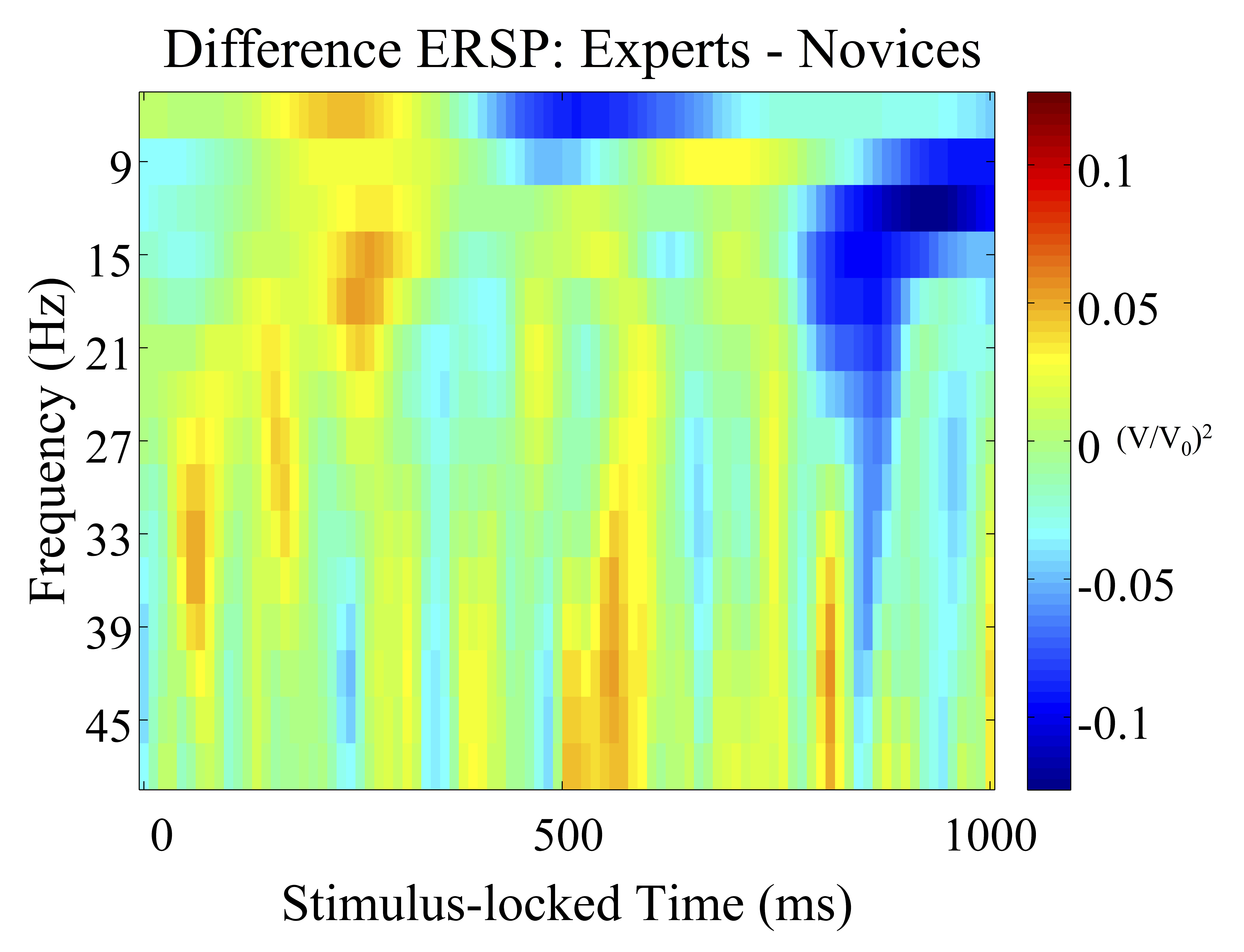

Supplement: S2 Fig — The difference between experts’ and novices’ mean differential activity are shown (experts—novices). (TIF) [file pone.0115629.s002.tif]
