# Supplementary material for: Experience Does Not Equal Expertise in Recognizing Infrequent Incoming Gunfire: Neural Markers for Experience and Task Expertise at Peak Behavioral Performance
Source: PLoS One. 2015 Feb 6;10(2):e0115629. doi: 10.1371/journal.pone.0115629 (PMC4319735; doi:10.1371/journal.pone.0115629)
Supplement: S1 Table — All other parameters set to FALSE besides those shown here. (DOCX) [file pone.0115629.s003.docx]

Table S1: sLORETA parameters used for source localization comparisons. All other parameters set to FALSE besides those shown here.

| **Within group sLORETA Parameters** | | **Between group sLORETA Parameters** | |
| --- | --- | --- | --- |
| **Name** | **Value** | **Name** | **Value** |
| No normalization | TRUE | No normalization | TRUE |
| Paired groups, test A=B | TRUE | Independent groups, test A=B | TRUE |
| No baseline | TRUE | No baseline | TRUE |
| All tests for all TimeFrames/Frequencies | TRUE | All tests for all TimeFrames/Frequencies | TRUE |
| t-statistic | TRUE | Log of ratio of averages | TRUE |
| Perform randomization SnPM | TRUE | Perform randomization SnPM | TRUE |
| Number of randomizations | 2000 | Number of randomizations | 2000 |

Left half of table shows parameters used to compare source distributions of TC and SC trials (paired t-tests within groups). Right half of table shows parameters used to compare source distributions of SC trials across groups (independent groups t-test).
