# Supplementary material for: Experience Does Not Equal Expertise in Recognizing Infrequent Incoming Gunfire: Neural Markers for Experience and Task Expertise at Peak Behavioral Performance
Source: PLoS One. 2015 Feb 6;10(2):e0115629. doi: 10.1371/journal.pone.0115629 (PMC4319735; doi:10.1371/journal.pone.0115629)
Supplement: S2 Table — (DOCX) [file pone.0115629.s004.docx]

Table S2: Experts’ MNI coordinates in mm and cortical structures showing greater neuronal source activity for TC trials than SC trials.

| **Response-Locked** | | | | | **Stimulus-Locked** | | | | |
| --- | --- | --- | --- | --- | --- | --- | --- | --- | --- |
| **X(MNI)** | **Y(MNI)** | **Z(MNI)** | **Voxel t-value** | **Structure** | **X(MNI)** | **Y(MNI)** | **Z(MNI)** | **Voxel t-value** | **Structure** |
| -5 | -15 | 55 | 4.924 | Medial Frontal Gyrus | 40 | 15 | 40 | 5.871 | Precentral Gyrus |
|  | | | | | 45 | 15 | 35 | 5.501 | Middle Frontal Gyrus |
|  |  |  |  |  | 50 | 15 | 40 | 5.490 | Middle Frontal Gyrus |
|  |  |  |  |  | 45 | 20 | 40 | 5.456 | Precentral Gyrus |
|  |  |  |  |  | 45 | 15 | 40 | 5.356 | Middle Frontal Gyrus |

All voxel t-values (paired t-test) are for p <= 0.01 and the result of correcting for multiple comparisons using statistical non-parametric mapping.
