# Supplementary material for: Experience Does Not Equal Expertise in Recognizing Infrequent Incoming Gunfire: Neural Markers for Experience and Task Expertise at Peak Behavioral Performance
Source: PLoS One. 2015 Feb 6;10(2):e0115629. doi: 10.1371/journal.pone.0115629 (PMC4319735; doi:10.1371/journal.pone.0115629)
Supplement: S3 Table — (DOCX) [file pone.0115629.s005.docx]

Table S3: Novices' MNI coordinates in mm and cortical structures showing greater neuronal source activity for TC trials than SC trials.

| **Response-Locked** | | | | | **Stimulus-Locked** | | | | |
| --- | --- | --- | --- | --- | --- | --- | --- | --- | --- |
| **X(MNI)** | **Y(MNI)** | **Z(MNI)** | **Voxel t-value** | **Structure** | **X(MNI)** | **Y(MNI)** | **Z(MNI)** | **Voxel t-value** | **Structure** |
| 50 | -35 | 45 | 7.508 | Inferior Parietal Lobule | -20 | -85 | 0 | 5.983 | Lingual Gyrus |
| 55 | -35 | 45 | 7.187 | Inferior Parietal Lobule | 15 | -80 | 0 | 5.930 | Lingual Gyrus |
| 15 | -35 | 50 | 6.971 | Paracentral Lobule | -30 | -95 | -10 | 5.918 | Inferior Occipital Gyrus |
| 45 | -35 | 45 | 6.919 | Inferior Parietal Lobule | -25 | -85 | -15 | 5.908 | Middle Occipital Gyrus |
| 5 | -35 | 45 | 6.777 | Precuneus | -50 | -60 | -25 | 5.837 | Fusiform Gyrus |
| -45 | -15 | 45 | 6.703 | Precentral Gyrus | -50 | -70 | -20 | 5.811 | Fusiform Gyrus |
| 15 | -35 | 45 | 6.690 | Cingulate Gyrus | -15 | -80 | 0 | 5.808 | Lingual Gyrus |
| 5 | -35 | 50 | 6.686 | Paracentral Lobule | -45 | -65 | -20 | 5.807 | Fusiform Gyrus |
| 45 | -35 | 40 | 6.672 | Inferior Parietal Lobule | -45 | -70 | -20 | 5.785 | Fusiform Gyrus |
| 15 | -35 | 55 | 6.633 | Paracentral Lobule | -45 | -60 | -25 | 5.774 | Fusiform Gyrus |
| 50 | -30 | 45 | 6.562 | Inferior Parietal Lobule | -30 | -90 | -20 | 5.752 | Inferior Occipital Gyrus |
| 15 | -40 | 70 | 6.430 | Postcentral Gyrus | -25 | -90 | -15 | 5.743 | Inferior Occipital Gyrus |
| 60 | -35 | 50 | 6.424 | Inferior Parietal Lobule | -20 | -80 | -10 | 5.717 | Lingual Gyrus |
| 45 | -30 | 45 | 6.423 | Postcentral Gyrus | -25 | -85 | -20 | 5.711 | Fusiform Gyrus |
| 0 | -35 | 45 | 6.392 | Paracentral Lobule | -50 | -60 | -20 | 5.682 | Fusiform Gyrus |
| 55 | -30 | 45 | 6.383 | Postcentral Gyrus | -25 | -80 | -15 | 5.667 | Lingual Gyrus |
| 50 | -35 | 40 | 6.346 | Inferior Parietal Lobule | -45 | -60 | -20 | 5.660 | Fusiform Gyrus |
| -50 | -15 | 40 | 6.273 | Precentral Gyrus | -30 | -85 | -20 | 5.638 | Fusiform Gyrus |
| -45 | -20 | 40 | 6.228 | Postcentral Gyrus | -40 | -65 | -20 | 5.628 | Fusiform Gyrus |
| 60 | -35 | 45 | 6.224 | Inferior Parietal Lobule | -50 | -65 | -15 | 5.613 | Middle Occipital Gyrus |
| -50 | -15 | 45 | 6.220 | Precentral Gyrus | -40 | -70 | -20 | 5.603 | Fusiform Gyrus |
| 55 | -35 | 50 | 6.205 | Postcentral Gyrus | -55 | -60 | -20 | 5.599 | Fusiform Gyrus |
| -60 | -10 | 40 | 6.202 | Precentral Gyrus | -25 | -80 | -20 | 5.584 | Fusiform Gyrus |
| -45 | -15 | 50 | 6.188 | Precentral Gyrus | -50 | -60 | -15 | 5.576 | Fusiform Gyrus |
| 5 | -35 | 55 | 6.167 | Paracentral Lobule | -25 | -90 | -20 | 5.555 | Inferior Occipital Gyrus |
| 20 | -35 | 45 | 6.112 | Cingulate Gyrus | -45 | -75 | -20 | 5.537 | Fusiform Gyrus |
| 45 | -30 | 40 | 6.106 | Postcentral Gyrus | -15 | -85 | 0 | 5.533 | Lingual Gyrus |
| 15 | -40 | 50 | 6.083 | Paracentral Lobule | -50 | -70 | -15 | 5.522 | Middle Occipital Gyrus |
| 0 | -35 | 50 | 6.063 | Paracentral Lobule | -55 | -60 | -15 | 5.517 | Inferior Temporal Gyrus |
| -40 | -20 | 40 | 6.044 | Precentral Gyrus | -15 | -75 | 0 | 5.515 | Lingual Gyrus |
| 50 | -30 | 40 | 5.986 | Postcentral Gyrus | -30 | -80 | -20 | 5.512 | Fusiform Gyrus |
| -5 | -35 | 45 | 5.986 | Precuneus | -65 | -45 | 20 | 5.505 | Superior Temporal Gyrus |
| 10 | -40 | 70 | 5.945 | Postcentral Gyrus | -50 | -60 | -10 | 5.492 | Middle Occipital Gyrus |
| 40 | -35 | 45 | 5.942 | Inferior Parietal Lobule | -30 | -85 | -15 | 5.490 | Middle Occipital Gyrus |
| 40 | -35 | 40 | 5.940 | Inferior Parietal Lobule | -50 | -55 | -25 | 5.488 | Fusiform Gyrus |
| -55 | -45 | 25 | 5.939 | Inferior Parietal Lobule | -40 | -60 | -20 | 5.481 | Fusiform Gyrus |
| 40 | -30 | 45 | 5.938 | Postcentral Gyrus | -55 | -65 | -15 | 5.476 | Middle Occipital Gyrus |
| 55 | -40 | 50 | 5.933 | Inferior Parietal Lobule | -20 | -80 | -15 | 5.469 | Lingual Gyrus |
| 50 | -35 | 50 | 5.930 | Postcentral Gyrus | -65 | -40 | 20 | 5.465 | Superior Temporal Gyrus |
| -20 | -20 | 40 | 5.924 | Cingulate Gyrus | -45 | -55 | -25 | 5.438 | Fusiform Gyrus |
| 50 | -40 | 45 | 5.911 | Inferior Parietal Lobule | -55 | -55 | -25 | 5.432 | Fusiform Gyrus |
| -40 | -30 | 30 | 5.910 | Postcentral Gyrus | -50 | -55 | -20 | 5.425 | Inferior Temporal Gyrus |
| -40 | -15 | 50 | 5.872 | Precentral Gyrus | -55 | -55 | -10 | 5.420 | Middle Temporal Gyrus |
| -15 | -25 | 40 | 5.848 | Cingulate Gyrus | 5 | -75 | 5 | 5.413 | Lingual Gyrus |
| 20 | -35 | 55 | 5.845 | Postcentral Gyrus | -35 | -70 | -20 | 5.412 | Fusiform Gyrus |
| -15 | -30 | 40 | 5.808 | Cingulate Gyrus | -55 | -60 | -10 | 5.410 | Inferior Temporal Gyrus |
| 5 | -35 | 40 | 5.801 | Cingulate Gyrus | -30 | -75 | -20 | 5.402 | Fusiform Gyrus |
| -40 | -30 | 35 | 5.790 | Postcentral Gyrus | -20 | -75 | -10 | 5.399 | Lingual Gyrus |
| 25 | -40 | 55 | 5.782 | Sub-Gyral | -55 | -55 | -15 | 5.398 | Middle Temporal Gyrus |
| -55 | -10 | 45 | 5.777 | Precentral Gyrus | -50 | -55 | -15 | 5.396 | Inferior Temporal Gyrus |
| 55 | -40 | 45 | 5.768 | Inferior Parietal Lobule | -55 | -55 | -20 | 5.391 | Fusiform Gyrus |
| 45 | -40 | 45 | 5.763 | Inferior Parietal Lobule | -50 | -55 | -10 | 5.382 | Inferior Temporal Gyrus |
| 60 | -30 | 45 | 5.751 | Postcentral Gyrus | -45 | -55 | -20 | 5.380 | Fusiform Gyrus |
| -15 | -30 | 45 | 5.750 | Cingulate Gyrus | -40 | -75 | -20 | 5.378 | Fusiform Gyrus |
| 0 | -35 | 40 | 5.742 | Cingulate Gyrus | -40 | -60 | -15 | 5.374 | Fusiform Gyrus |
| -10 | -30 | 45 | 5.739 | Cingulate Gyrus | -50 | -60 | -5 | 5.369 | Inferior Temporal Gyrus |
| -10 | -30 | 40 | 5.735 | Cingulate Gyrus | -25 | -75 | -20 | 5.365 | Fusiform Gyrus |
| -40 | -20 | 45 | 5.708 | Precentral Gyrus | 10 | -75 | 0 | 5.359 | Lingual Gyrus |
| 40 | -30 | 40 | 5.690 | Postcentral Gyrus | -25 | -75 | -10 | 5.351 | Lingual Gyrus |
| 5 | -40 | 45 | 5.676 | Cingulate Gyrus | -45 | -60 | -5 | 5.350 | Middle Temporal Gyrus |
| -45 | -20 | 45 | 5.669 | Precentral Gyrus | -60 | -55 | -10 | 5.329 | Inferior Temporal Gyrus |
| -20 | -30 | 40 | 5.658 | Cingulate Gyrus | -45 | -55 | -15 | 5.318 | Fusiform Gyrus |
| 45 | -30 | 50 | 5.655 | Postcentral Gyrus | -20 | -70 | 5 | 5.306 | Posterior Cingulate |
| -45 | -30 | 30 | 5.653 | Inferior Parietal Lobule | 15 | -85 | 0 | 5.303 | Lingual Gyrus |
| -5 | -35 | 40 | 5.643 | Cingulate Gyrus | -60 | -55 | -15 | 5.300 | Inferior Temporal Gyrus |
| 15 | -35 | 40 | 5.640 | Cingulate Gyrus | -50 | -65 | -5 | 5.297 | Inferior Temporal Gyrus |
| -50 | -20 | 40 | 5.635 | Postcentral Gyrus | -45 | -60 | 0 | 5.288 | Middle Temporal Gyrus |
| -5 | -30 | 45 | 5.631 | Cingulate Gyrus | -20 | -80 | -20 | 5.284 | Fusiform Gyrus |
| 15 | -40 | 65 | 5.621 | Postcentral Gyrus | -45 | -65 | -5 | 5.278 | Middle Temporal Gyrus |
| -40 | -25 | 40 | 5.618 | Postcentral Gyrus | -20 | -70 | 10 | 5.276 | Cuneus |
| 50 | -30 | 50 | 5.614 | Inferior Parietal Lobule | -35 | -90 | -20 | 5.271 | Inferior Occipital Gyrus |
| -55 | -15 | 40 | 5.613 | Precentral Gyrus | 15 | -75 | 0 | 5.262 | Lingual Gyrus |
| 5 | -35 | 60 | 5.609 | Paracentral Lobule | -60 | -55 | -20 | 5.262 | Inferior Temporal Gyrus |
| 45 | -35 | 50 | 5.601 | Inferior Parietal Lobule | -20 | -75 | -15 | 5.262 | Lingual Gyrus |
| -5 | -30 | 40 | 5.573 | Cingulate Gyrus | -60 | -60 | -10 | 5.261 | Inferior Temporal Gyrus |
| 5 | -30 | 45 | 5.572 | Cingulate Gyrus | -60 | -55 | -5 | 5.259 | Middle Temporal Gyrus |
| 0 | -30 | 45 | 5.571 | Paracentral Lobule | -20 | -85 | -20 | 5.256 | Fusiform Gyrus |
| 0 | -35 | 55 | 5.566 | Paracentral Lobule | 10 | -80 | -5 | 5.254 | Lingual Gyrus |
| 10 | -30 | 45 | 5.565 | Cingulate Gyrus | -15 | -75 | -5 | 5.249 | Lingual Gyrus |
| -45 | -30 | 35 | 5.559 | Postcentral Gyrus | -40 | -55 | -25 | 5.248 | Fusiform Gyrus |
| 10 | -40 | 50 | 5.547 | Paracentral Lobule | -55 | -65 | -10 | 5.246 | Middle Occipital Gyrus |
| 55 | -30 | 50 | 5.543 | Postcentral Gyrus | 60 | -30 | 25 | 5.243 | Inferior Parietal Lobule |
| -5 | -35 | 50 | 5.529 | Paracentral Lobule | -15 | -75 | 10 | 5.238 | Cuneus |
| -45 | -25 | 20 | 5.516 | Insula | -35 | -80 | -20 | 5.230 | Fusiform Gyrus |
| -50 | -15 | 50 | 5.516 | Postcentral Gyrus | -60 | -40 | 20 | 5.227 | Superior Temporal Gyrus |
| -40 | -25 | 20 | 5.508 | Insula | -40 | -55 | -20 | 5.225 | Fusiform Gyrus |
| -45 | -25 | 40 | 5.506 | Postcentral Gyrus | -20 | -70 | 0 | 5.221 | Lingual Gyrus |
| -50 | -10 | 50 | 5.506 | Precentral Gyrus | -20 | -75 | 10 | 5.213 | Cuneus |
| 50 | -40 | 50 | 5.505 | Inferior Parietal Lobule | -35 | -85 | -20 | 5.183 | Inferior Occipital Gyrus |
| -15 | -25 | 45 | 5.504 | Cingulate Gyrus | 5 | -80 | 5 | 5.171 | Lingual Gyrus |
| 60 | -40 | 45 | 5.503 | Inferior Parietal Lobule | -55 | -70 | -10 | 5.170 | Middle Occipital Gyrus |
| 45 | -35 | 35 | 5.490 | Inferior Parietal Lobule | 15 | -75 | -5 | 5.164 | Lingual Gyrus |
| -40 | -35 | 35 | 5.485 | Inferior Parietal Lobule | -30 | -70 | -15 | 5.162 | Fusiform Gyrus |
| 15 | -30 | 45 | 5.477 | Cingulate Gyrus | 5 | -75 | 0 | 5.146 | Lingual Gyrus |
| -15 | -20 | 40 | 5.469 | Cingulate Gyrus | -25 | -70 | -15 | 5.145 | Fusiform Gyrus |
| -20 | -30 | 45 | 5.469 | Cingulate Gyrus | -20 | -70 | -5 | 5.139 | Lingual Gyrus |
| 20 | -35 | 60 | 5.458 | Postcentral Gyrus | 20 | -80 | -10 | 5.115 | Lingual Gyrus |
| 45 | -50 | 55 | 5.457 | Inferior Parietal Lobule | -15 | -80 | -10 | 5.099 | Lingual Gyrus |
| 60 | -30 | 50 | 5.444 | Postcentral Gyrus | -15 | -70 | 0 | 5.099 | Lingual Gyrus |
| 0 | -30 | 40 | 5.442 | Cingulate Gyrus | 15 | -80 | -10 | 5.097 | Lingual Gyrus |
| 30 | -45 | 60 | 5.436 | Postcentral Gyrus | -55 | -60 | -5 | 5.091 | Middle Temporal Gyrus |
| 40 | -40 | 40 | 5.435 | Inferior Parietal Lobule | 20 | -85 | 0 | 5.087 | Lingual Gyrus |
| -20 | -20 | 45 | 5.434 | Cingulate Gyrus | -45 | -65 | 0 | 5.083 | Middle Temporal Gyrus |
| 15 | -40 | 55 | 5.417 | Paracentral Lobule | -10 | -75 | 0 | 5.073 | Lingual Gyrus |
| 20 | -35 | 40 | 5.415 | Cingulate Gyrus | -20 | -70 | -10 | 5.069 | Lingual Gyrus |
| 10 | -40 | 65 | 5.414 | Postcentral Gyrus | 10 | -75 | -5 | 5.066 | Lingual Gyrus |
| -55 | -15 | 45 | 5.397 | Postcentral Gyrus | -50 | -70 | -5 | 5.065 | Inferior Temporal Gyrus |
| 5 | -30 | 40 | 5.372 | Cingulate Gyrus | -40 | -80 | -20 | 5.054 | Fusiform Gyrus |
| 15 | -35 | 70 | 5.371 | Precentral Gyrus |  | | | | |
| -50 | -25 | 35 | 5.368 | Postcentral Gyrus |  |  |  |  |  |
| -40 | -20 | 15 | 5.367 | Insula |  |  |  |  |  |
| -45 | -30 | 20 | 5.366 | Insula |  |  |  |  |  |
| -10 | -25 | 40 | 5.366 | Cingulate Gyrus |  |  |  |  |  |
| 35 | -30 | 45 | 5.366 | Postcentral Gyrus |  |  |  |  |  |
| -15 | -35 | 40 | 5.366 | Cingulate Gyrus |  |  |  |  |  |
| -35 | -25 | 40 | 5.358 | Postcentral Gyrus |  |  |  |  |  |
| 55 | -45 | 50 | 5.352 | Inferior Parietal Lobule |  |  |  |  |  |
| 10 | -30 | 40 | 5.334 | Cingulate Gyrus |  |  |  |  |  |
| 5 | -40 | 40 | 5.331 | Cingulate Gyrus |  |  |  |  |  |
| -50 | -50 | 25 | 5.325 | Supramarginal Gyrus |  |  |  |  |  |
| 40 | -35 | 35 | 5.321 | Inferior Parietal Lobule |  |  |  |  |  |
| 0 | -40 | 45 | 5.313 | Precuneus |  |  |  |  |  |
| 40 | -40 | 45 | 5.304 | Inferior Parietal Lobule |  |  |  |  |  |
| 40 | -30 | 50 | 5.301 | Postcentral Gyrus |  |  |  |  |  |
| -15 | -35 | 45 | 5.301 | Cingulate Gyrus |  |  |  |  |  |
| 25 | -40 | 60 | 5.301 | Sub-Gyral |  |  |  |  |  |
| 35 | -30 | 40 | 5.300 | Sub-Gyral |  |  |  |  |  |
| 20 | -30 | 45 | 5.296 | Cingulate Gyrus |  |  |  |  |  |
| 15 | -30 | 40 | 5.288 | Cingulate Gyrus |  |  |  |  |  |
| -50 | -10 | 45 | 5.288 | Precentral Gyrus |  |  |  |  |  |
| 20 | -40 | 60 | 5.279 | Postcentral Gyrus |  |  |  |  |  |
| 45 | -10 | 10 | 5.272 | Insula |  |  |  |  |  |
| -40 | -15 | 15 | 5.265 | Insula |  |  |  |  |  |
| 25 | -35 | 50 | 5.245 | Postcentral Gyrus |  |  |  |  |  |
| -60 | -40 | 25 | 5.243 | Inferior Parietal Lobule |  |  |  |  |  |
| 20 | -40 | 70 | 5.231 | Postcentral Gyrus |  |  |  |  |  |
| 35 | -25 | 45 | 5.230 | Postcentral Gyrus |  |  |  |  |  |
| 45 | -15 | 15 | 5.224 | Insula |  |  |  |  |  |
| -55 | -15 | 35 | 5.219 | Precentral Gyrus |  |  |  |  |  |
| 20 | -30 | 40 | 5.214 | Cingulate Gyrus |  |  |  |  |  |
| -35 | -30 | 40 | 5.191 | Sub-Gyral |  |  |  |  |  |
| -40 | -30 | 40 | 5.189 | Postcentral Gyrus |  |  |  |  |  |
| -40 | -20 | 50 | 5.186 | Precentral Gyrus |  |  |  |  |  |
| 45 | -30 | 35 | 5.184 | Postcentral Gyrus |  |  |  |  |  |
| -45 | -35 | 35 | 5.182 | Inferior Parietal Lobule |  |  |  |  |  |
| 15 | -40 | 60 | 5.178 | Paracentral Lobule |  |  |  |  |  |
| -20 | -35 | 40 | 5.175 | Cingulate Gyrus |  |  |  |  |  |
| 40 | -25 | 45 | 5.172 | Postcentral Gyrus |  |  |  |  |  |
| 45 | -20 | 15 | 5.171 | Insula |  |  |  |  |  |
| 45 | -50 | 50 | 5.169 | Inferior Parietal Lobule |  |  |  |  |  |
| 45 | -55 | 55 | 5.160 | Inferior Parietal Lobule |  |  |  |  |  |
| 50 | -45 | 50 | 5.160 | Inferior Parietal Lobule |  |  |  |  |  |
| -40 | -15 | 10 | 5.150 | Insula |  |  |  |  |  |
| 0 | -35 | 60 | 5.149 | Paracentral Lobule |  |  |  |  |  |
| 55 | -35 | 40 | 5.146 | Inferior Parietal Lobule |  |  |  |  |  |
| -55 | -20 | 40 | 5.127 | Postcentral Gyrus |  |  |  |  |  |
| 50 | -50 | 55 | 5.119 | Inferior Parietal Lobule |  |  |  |  |  |
| -45 | -25 | 15 | 5.118 | Insula |  |  |  |  |  |
| 45 | -15 | 10 | 5.117 | Insula |  |  |  |  |  |
| 40 | -30 | 35 | 5.103 | Postcentral Gyrus |  |  |  |  |  |
| 45 | -40 | 50 | 5.090 | Inferior Parietal Lobule |  |  |  |  |  |
| 60 | -45 | 45 | 5.086 | Inferior Parietal Lobule |  |  |  |  |  |
| -60 | -15 | 40 | 5.085 | Precentral Gyrus |  |  |  |  |  |
| -5 | -35 | 55 | 5.075 | Paracentral Lobule |  |  |  |  |  |
| -55 | -25 | 40 | 5.072 | Postcentral Gyrus |  |  |  |  |  |
| -40 | -30 | 20 | 5.070 | Insula |  |  |  |  |  |
| -5 | -40 | 40 | 5.068 | Cingulate Gyrus |  |  |  |  |  |

All voxel t-values (paired t-test) are for p <= 0.01 and the result of correcting for multiple comparisons using statistical non-parametric mapping.
