# Supplementary material for: Experience Does Not Equal Expertise in Recognizing Infrequent Incoming Gunfire: Neural Markers for Experience and Task Expertise at Peak Behavioral Performance
Source: PLoS One. 2015 Feb 6;10(2):e0115629. doi: 10.1371/journal.pone.0115629 (PMC4319735; doi:10.1371/journal.pone.0115629)
Supplement: S4 Table — (DOCX) [file pone.0115629.s006.docx]

Table S4: MNI coordinates in mm and cortical structure showing greater neuronal source activity for experts than novices during SC trials.

| **Response-Locked** | | | | |
| --- | --- | --- | --- | --- |
| **X(MNI)** | **Y(MNI)** | **Z(MNI)** | **Voxel t-value** | **Structure** |
| -55 | -50 | 25 | 2.354 | Supramarginal Gyrus |

Voxel t-value (applied to log f-ratio, independent groups t-test) is for p < 0.05 and the result of correcting for multiple comparisons using statistical non-parametric mapping.
